# Chiroptical Synaptic Heterojunction Phototransistors Based on Self‐Assembled Nanohelix of π‐Conjugated Molecules for Direct Noise‐Reduced Detection of Circularly Polarized Light

**DOI:** 10.1002/advs.202304039

**Published:** 2023-07-27

**Authors:** Hanna Lee, Jun Ho Hwang, Seung Ho Song, Hyemi Han, Seo‐Jung Han, Bong Lim Suh, Kahyun Hur, Jihoon Kyhm, Jongtae Ahn, Jeong Ho Cho, Do Kyung Hwang, Eunji Lee, Changsoon Choi, Jung Ah Lim

**Affiliations:** ^1^ Center for Opto‐Electronic Materials and Devices Korea Institute of Science and Technology Seoul 02792 Republic of Korea; ^2^ Department of Chemical and Biomolecular Engineering Yonsei University Seoul 03722 Republic of Korea; ^3^ School of Materials Science and Engineering Gwangju Institute of Science and Technology Gwangju 61005 Republic of Korea; ^4^ Chemical and Biological Integrative Research Center Korea Institute of Science and Technology Seoul 02792 Republic of Korea; ^5^ Division of Bio‐Medical Science and Technology KIST School University of Science and Technology of Korea Seoul 02792 Republic of Korea; ^6^ Extreme Materials Research Center Korea Institute of Science and Technology Seoul 02792 Republic of Korea; ^7^ Technology Support Center Korea Institute of Science and Technology Seoul 02792 Republic of Korea; ^8^ KU‐KIST Graduate School of Converging Science and Technology Korea University Seoul 02841 Republic of Korea; ^9^ Division of Nano and Information Technology KIST School University of Science and Technology Seoul 02792 Republic of Korea

**Keywords:** chiral‐optoelectronics, circularly polarized light, nanohelix, self‐assembly of conjugated molecule, synaptic photodetector

## Abstract

High‐performance chiroptical synaptic phototransistors are successfully demonstrated using heterojunctions composed of a self‐assembled nanohelix of a π‐conjugated molecule and a metal oxide semiconductor. To impart strong chiroptical activity to the device, a diketopyrrolopyrrole‐based π‐conjugated molecule decorated with chiral glutamic acid is newly synthesized; this molecule is capable of supramolecular self‐assembly through noncovalent intermolecular interactions. In particular, nanohelix formed by intertwinded fibers with strong and stable chiroptical activity in a solid‐film state are obtained through hydrogen‐bonding‐driven, gelation‐assisted self‐assembly. Phototransistors based on interfacial charge transfer at the heterojunction from the chiroptical nanohelix to the metal oxide semiconductor show excellent chiroptical detection with a high photocurrent dissymmetry factor of 1.97 and a high photoresponsivity of 218 A W^−1^. The chiroptical phototransistor demonstrates photonic synapse‐like, time‐dependent photocurrent generation, along with persistent photoconductivity, which is attributed to the interfacial charge trapping. Through the advantage of synaptic functionality, a trained convolutional neural network successfully recognizes noise‐reduced circularly polarized images of handwritten alphabetic characters with better than 89.7% accuracy.

## Graphical abstract

High‐performance chiroptical synaptic phototransistors are demonstrated using a heterojunction composed of a self‐assembled nanohelices of chiral π‐conjugated molecules and metal oxide semiconductor. The phototransistors based on interfacial charge transfer from the chiroptical nanohelices to the metal oxide semiconductor show excellent chiroptical sensing capability and time‐dependent photocurrent generation behavior like photonic synapse for neural‐network‐based image recognition of circularly polarized light.

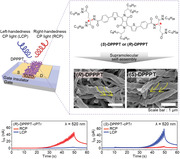

## 1 Introduction

Chiral optoelectronic devices, which communicate information through circularly polarized (CP) light, have attracted significant attention because of their broad potential encompassing electron spin filtering, enantiopure chiral magnets, chiral imaging, quantum optics, spintronics, chiral biosensing, and cryptography.^[^ ^1^, ^2^, ^3^, ^4^, ^5^, ^6^ ^]^ In particular, a chiroptical photodetector that converts the circular polarization direction and intensity of CP light into identifiable electrical signals is one of the key components that link chiroptical information to various integrated electronics systems. Recently, substantial progress has been made in developing CP light detecting optoelectronic devices such as photodiodes and phototransistors through the use of various chiral nanomaterials such as plasmonic meta‐materials,^[^ ^7^, ^8^ ^]^ organic–inorganic hybrid perovskites,^[^ ^9^, ^10^ ^]^ π‐conjugated molecules,^[^ ^11^, ^12^ ^]^ cellulose,^[^ ^13^ ^]^ and cholesteric liquid crystals^[^ ^14^ ^]^ that exhibit orientation‐selective light–matter interaction with CP light. This research has paved a promising path to overcome the current limitations of CP light detection, which relies on bulky and complicated optical components, including linear polarizers and quarter‐wave plates.^[^ ^15^, ^16^ ^]^ However, from a practical perspective of integration with existing optoelectronic circuit systems, there is still a need to develop a high‐performance chiroptical photodetector that demonstrates high photoresponsivity and sensitivity, excellent electrical signal identification of the polarization direction of CP light, simple fabrication processability, and feasibility for device integration.

Among various chiral nanomaterials for optoelectronic devices, π‐conjugated molecules are one of the most promising candidates to meet the above requirements.^[^ ^11^, ^12^ ^]^ The electronic energy structure and chiroptical properties of chiral π‐conjugated molecules can be finely tuned according to the molecular design and aggregate forms.^[^ ^17^ ^]^ Moreover, the self‐assembly of chiral π‐conjugated molecules provide a simple and promising approach to amplify chiroptical activities or to transfer optical chirality to other light‐absorption ranges for various applications.^[^ ^18^, ^19^, ^20^ ^]^ However, from the viewpoint of high‐performance chiroptical photodetectors, achieving such strong chiroptical sensitivity while maintaining excellent photocurrent generation and charge‐transport properties in the photoactive channel based on chiral π‐conjugated molecules remains a challenge. Chiroptical interactions in π‐conjugated molecules are intrinsically achieved by breaking the symmetry of the atomic or molecular orbital distributions. This may affect the molecular rearrangement and degrade their charge‐transport characteristics.^[^ ^21^ ^]^ For example, the helical chiral molecule 1‐aza[6]helicene, a representative chiroptical organic semiconductor, exhibits strong circular dichroism (CD) because of its twisted π‐conjugated orbitals. However, the field‐effect mobility of enantiopure 1‐aza[6]helicene was found to be 80‐fold lower than that of the chiral racemic mixture.^[^ ^21^ ^]^ Therefore, a new strategy is needed to extend the utilization of chiral π‐conjugated molecules to high‐performance chiral optoelectronic devices.

As a viable alternative, heterojunction devices consisting of chiral nanomaterial and semiconductors offer an effective approach for developing high‐performance chiral optoelectronic devices. In operation principle, photogenerated charge carriers at a chiral π‐conjugated molecule by selectively absorbing CP light can be transferred to the semiconductor layer at the heterojunction interface, leading to a current increase through the efficient charge transport in the semiconductor layer. This approach enables independent management and optimization of the charge transport behavior of the semiconductors and the chiroptical properties of the nanomaterials. In fact, chiral hot‐electron devices are exemplified, where the plasmonic hot electrons generated by CP light at chiral nanoparticles or nanopatterns are transferred to silicon,^[^ ^22^ ^]^ a metal oxide (e.g., InGaZnO),^[^ ^23^ ^]^ or a perovskite‐type material,^[^ ^24^ ^]^ generating a chiroptical‐sensitive photocurrent. Recently, chiroptical photodetectors fabricated by integrating chiral supramolecular polymers with organic field‐effect transistors (e.g., poly(3‐hexylthiophene‐2,5‐diyl) (P3HT)) have shown improved chiroptical sensitivity compared with those fabricated with conventional π‐conjugated molecules.^[^ ^25^ ^]^ Although many studies of high‐performance typical photodetectors (i.e., no chiroptical response) based on heterojunctions of various nanomaterials (e.g., organic–inorganic,^[^ ^26^ ^]^ 2D materials,^[^ ^27^ ^]^ quantum dot‐InGaZnO (IGZO)^[^ ^28^ ^]^) have been reported, the literature contains few examples of the implementation of chiroptical photodetectors that utilize such heterojunction charge transfer.

A more attractive aspect of this heterojunction photodetection approach is that control of charge trapping behavior at the interface can confer synapse‐inspired optoelectronic properties, such as time‐dependent photocurrent generation and persistent photoconductivity (PPC), to the photodetectors.^[^ ^29^, ^30^ ^]^ Recently, synaptic photodetectors with such characteristics have shown promise for accurately and efficiently perceiving light signals.^[^ ^31^ ^]^ During the image‐sensing step, synaptic photodetectors perform signal preprocessing, such as contrast enhancement and noise filtering, without requiring additional computations.^[^ ^32^, ^33^, ^34^ ^]^ This preprocessing reduces the computational burden in the signal recognition step compared with that in the established architecture of a front‐end sensor and back‐end processor. In addition, the preprocessed signals can be accurately recognized by an artificial neural network.^[^ ^35^, ^36^ ^]^ With these advantages, synaptic photodetection can also enhance the detection accuracy of CP light. However, attempts to develop chiroptical photodetectors with synaptic functionality are still in the beginning stages. Only synaptic photoresponses in chiroptical heterojunction phototransistors based on hot‐electron transfer of chiral nanoparticles have thus far been demonstrated.^[^ ^23^ ^]^ Groundbreaking advances in high‐performance chiral optoelectronics will require further research efforts to extend this strategy of chiroptical synaptic devices to various chiral nanomaterials.

Herein, for improved detection accuracy of CP light, we propose a chiroptical synaptic heterojunction phototransistor based on a self‐assembled nanohelix of π‐conjugated molecules. To take advantage of chiral π‐conjugated molecules, we synthesized a diketopyrrolopyrrole (DPP)‐based chiral molecule capable of forming self‐assembled supramolecular aggregates with strong chiroptical absorption. We chose DPP as the core conjugated building block because of its tunable absorption spectrum, enhancing backbone planarity with incorporation of noncovalent conformational locks, cost‐effective processability, and good batch‐to‐batch repeatability.^[^ ^37^, ^38^, ^39^ ^]^ Introduction of alkylated chiral glutamic acid to the donor‐acceptor type DPP‐conjugated backbone enabled various noncovalent intermolecular interactions, including π–π interaction, hydrogen bonding, and solvophobic interaction. The self‐assembled aggregates of the DPP chiral molecule exhibited various nanoarchitectures depending on the controlled intermolecular interactions. Especially, the intertwined nanohelix fibers formed by gelation exhibited strong chiroptical absorption in both the solution and solidified film states. Deposition of the DPP‐based nanohelix fibers onto an amorphous‐IGZO transistor led to a chiroptical heterojunction phototransistor capable of distinguishing CP light with different rotation orientations because of the synergistic effect of the nanohelix fibers' strong chiroptical activity and IGZO's high electron mobility. On the basis of the time‐dependent photocurrent generation and PPC of the device, neural‐network‐based image recognition using CP light was demonstrated to achieve highly accurate polarization‐based selective detection, even for noisy images.

## 2 Results and Discussion

**Figure 1:**
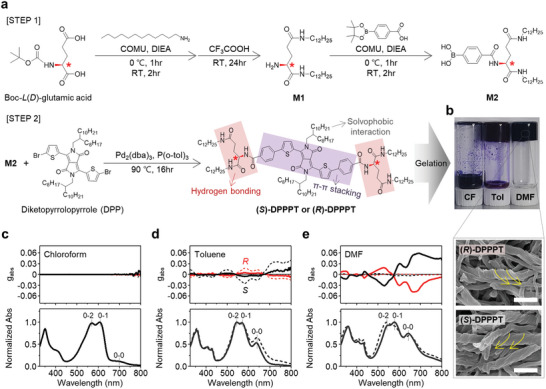
a) Synthetic route to DPP‐based chiral organogelators (DPPPT) designed to allow diverse noncovalent intermolecular interactions. b) The photograph shows the capability to form the DPPPT organogels formed in chloroform (CF, left), toluene (Tol, middle), and DMF (right) by gelation‐assisted self‐assembly. SEM images of (*R*)‐DPPPT (top) and (*S*)‐DPPPT (bottom) gels formed in DMF (scale bar = 1 µm). c–e) Dissymmetry factors (*g*_abs_) and normalized absorption spectra of (*R*)‐DPPPT (red line) and (*S*)‐DPPPT (black line) dispersions in organic solvents c) chloroform, d) toluene, and e) DMF. The dashed and solid lines indicate before and after gelation, respectively.

**Figure** **1**a shows the synthesis route to DPP‐based chiral organogelators, 2,2′‐((4,4′‐((2,5‐bis(2‐octyldodecyl)‐3,6‐dioxo‐2,3,5,6‐tetrahydropyrrolo[3,4‐*c*]pyrrole‐1,4‐diyl)bis(thiophene‐5,2‐diyl))bis(benzoyl))bis(azanediyl))bis(*N* ^1^,*N* ^5^‐didodecylpentanediamide) (DPPPT). First, 2‐amino‐*N* ^1^,*N* ^5^‐didodecylpentanediamide (M1) was prepared via a (1‐cyano‐2‐ethoxy‐2‐oxoethylidenaminooxy)dimethylamino‐morpholino‐carbenium hexafluorophosphate (COMU) coupling reaction of the amine‐protected glutamic acid with *tert*‐butyl carbamates (Boc) (e.g., Boc‐( *l* )‐glutamic acid and Boc‐( *d* )‐glutamic acid) and dodecylamine. After deprotection of the Boc‐group, (4‐((1,5‐bis(dodecylamino)‐1,5‐dioxopentan‐2‐yl)carbamoyl)phenyl)boronic acid (M2) was prepared by adding 4‐carboxylphenylboronic acid pinacol ester to the secondary amine of M1 via a COMU coupling reaction. Finally, (*S*)‐DPPPT or (*R*)‐DPPPT was obtained through a Suzuki–Miyaura coupling reaction between thiophene‐flanked diketopyrrolopyrrole (DPP) and M2. Additional details of the synthesis procedure are provided in Figure S1 and Method S1, Supporting Information. The successful synthesis of DPPPT was confirmed by ^1^H‐nuclear magnetic resonance (^1^H‐NMR) spectroscopy and matrix‐assisted laser desorption/ionization time‐of‐flight (MALDI‐TOF) mass spectrometry (Method S1, Supporting Information).

The molecular structure of DPPPT was designed to allow diverse noncovalent intermolecular interactions such as π–π stacking, hydrogen bonding, and solvophobic interactions for the DPP‐conjugated core, the functionalized chiral amide groups, and the alkyl chains, respectively (Figure 1a). These interactions can act as driving forces for the self‐assembly of DPPPTs into one‐dimensional nanostructures that facilitate the gelation of DPPPT aggregates in organic solvents with unique chiroptical activities.^[^ ^40^, ^41^, ^42^ ^]^ To induce gelation‐assisted self‐assembly, DPPPT was dissolved in various organic solvents with different solubility, such as chloroform, toluene, and *N*,*N*‐dimethylformamide (DMF), at the boiling point of each solvent for 10 min and then cooled to room temperature (RT). As shown in the photograph in Figure 1b, DPPPT organogels were formed in the DMF and toluene but not in chloroform. It is noted that the organogel prepared using DMF was stable and did not flow over time, which differed from that prepared in toluene. Figure 1b shows scanning electron microscopy (SEM) images of the nanostructures of the DMF‐based DPPPT organogel. Interestingly, (*R*)‐DPPPT and (*S*)‐DPPPT formed right‐handed and left‐handed nanohelixes, respectively, indicating that molecular chirality can be transferred to supramolecular assemblies. A more detailed discussion of the self‐assembly of DPPPT as a function of solvents is explained below.

Figure 1c–e shows the UV–vis absorption and circular dichroism (CD) spectra of DPPPT solutions (0.08 mg mL^−1^). The optical absorption spectrum of the DPPPT monolayer, as calculated by density function theory (DFT), shows peaks characteristic of π–π* (≈348 nm), 0–2 (≈570 nm), and 0–1 (≈607 nm) transitions^[^ ^43^, ^44^ ^]^ (Figure S2, Supporting Information). In chloroform, DPPPTs are molecularly well dissolved; thus, such characteristic absorptions were observed (Figure 1c), consistent with the previously reported absorption behavior of thiophene‐flanked DPP molecules with intramolecular charge transfer from the donor to the acceptor unit in the DPP molecular structure.^[^ ^43^, ^44^, ^45^ ^]^ The red‐shifted π–π* transition and appearance of weak 0–0 transitions at ≈700 nm suggest a small amount of J‐aggregate compared to toluene and DMF.^[^ ^46^, ^47^ ^]^ Notably, no chiroptical response (i.e., dissymmetry factor for absorption, *g*_abs_) was observed. By contrast, DPPPTs dissolved in toluene (Figure 1d) or DMF (Figure 1e) showed an enhanced 0–0 transition and blue‐shifted 0–2 and 0–1 transitions compared with the chloroform (Figure S3, Supporting Information) while exhibiting definite symmetric CD signals at such main absorptions according to the chiral preferred handedness of DPPPT. These results indicate that the chiroptically self‐assembly of DPPPT molecules is driven by the marginal solubility of DPPPT in toluene or DMF. According to Kasha's exciton theory, the changes in absorption spectra observed in the self‐assembly of DPPPT are attributed to the joint H‐ (i.e., face‐to‐face arrangement) and J‐type (i.e., head‐to‐tail arrangement) aggregation behaviors.^[^ ^48^ ^]^ The presence of bisignate CD responses at the characteristic absorption maximum, known as ‘Cotton effect’, indicates the existence of an exciton couplet because of the twisted alignment of the transition dipole moment.^[^ ^17^ ^]^ In particular, the CD spectrum of the DMF‐based DPPPT aggregates shows a substantial enhancement of the CD signal, along with a hypsochromic shift in the characteristic absorptions, after gelation‐assisted assembly. This result suggests that the twisted face‐to‐face stacking of the DPPPT core occurred during the gelation process, resulting in strong chiroptical absorption. By contrast, the DPPPT aggregates prepared using toluene showed a decrease in *g*_abs_ and weaker 0–0 absorption after the gelation process, suggesting that DPPPT aggregates in toluene are assembled differently into an entangled supramolecular structure through molecular interaction with the solvent.

**Figure 2:**
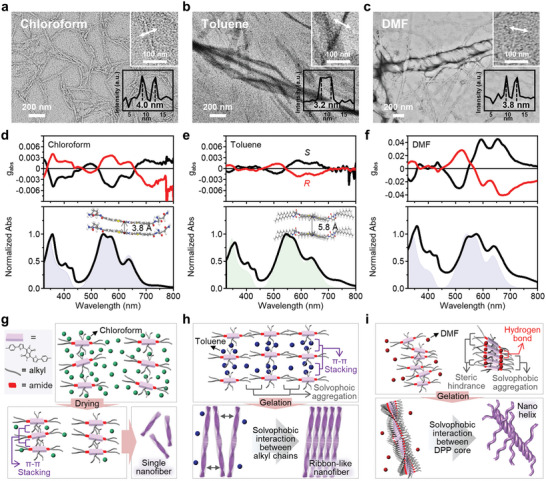
a–c) Negative stained TEM images of 1D DPPPT fiber, ribbon‐like, and coiled coil‐like assemblies formed in a) chloroform, b) toluene, and c) DMF, respectively. The insets show magnified TEM images of elemental fibers capable of the hierarchical assembly and intensity profiles obtained from the double‐headed arrows marked in white in the vertical direction of the fibers in the inset. d–f) Dissymmetry factor (*g*_abs_) and normalized absorption spectrum of thin films of (*R*)‐DPPPT (red line) and (*S*)‐DPPPT (black line). The thin films were formed by drop‐casting the gels dispersed in d) chloroform, e) toluene, and f) DMF. The blue regions in (d) and (f) indicate the absorption spectra of DPPPT dimers simulated by DFT using a simplified molecular structure without an alkyl side chain on the DPP core; the green region in (e) indicates the DFT‐simulated spectral absorption of original DPPPT dimers. g–i) Schematics showing the formation process of the self‐assembled DPPPTs in g) chloroform, h) toluene, and i) DMF, respectively.

To gain a deeper understanding of the aggregation mechanism of DPPPT, we conducted further studies on the supramolecular structure and optical properties of the gelation‐assisted DPPPT assemblies. **Figure** **2**a–c shows transmission electron microscopy (TEM) images of the drop‐cast thin films of the DPPPT assemblies negatively stained with uranyl acetate, revealing that different nanoarchitectures of DPPPT were formed depending on the solvent utilized. The homogeneously distributed nanofibers with a width of ≈20 nm, in which the fibers with a width of ≈4 nm were bundled, were observed in chloroform solution (Figure 2a). Given that the size of the long axis of the molecule crossing the conjugated backbone is ≈3.9 nm calculated by CPK modeling (Figure S4, Supporting Information). In order to verify the experimental results of intermolecular distance, we calculated the optimized structures in the DPPPT dimers caused by π–π stacking utilizing the DFT calculations (detailed in the Section 4). The alkyl side chains in the DPP were removed from the initial optimized periodic structures to simplify the stacking configurations (Figure 2d). The optimized simulated intermolecular distance between π–π stacked molecules was 3.8 Å (Figure 2d and Figure S5a, Supporting Information), consistent with the distance empirically measured (3.8 Å) using the selected area electron diffraction (SAED) pattern of TEM (Figure S6a, Supporting Information). These observations support our speculation that enhanced π–π interaction of the DPP‐conjugated backbone during solvent evaporation leads to preferential face‐to‐face stacking of the DPPPT molecules along the long nanofiber axis (Figure 2g). Notably, the DPPPT thin films composed of such nanofibers exhibit weak chiroptical activity (Figure 2d), in contrast to the absence of chiroptical activity observed for the chloroform‐based solution (Figure 1c). In toluene, which is a poor solvent for the alkyl side chains of DPPPT, a ribbon‐like structure composed of parallelly aligned single nanofibers was formed (Figure 2b). The inset of TEM image in Figure 2b shows that the edge of the elemental ribbon‐like nanofibers is ≈3.2 nm which often is observed. The limited solubility of DPPPT can increase the solvophobic interaction between the alkyl groups on the surface of individual nanofibers, which occurs during the evaporation of toluene, resulting in parallel assembly of the DPPPT nanofibers (Figure 2h). The optical absorption spectrum of the toluene‐gelated DPPPT film mostly well‐matched the DFT‐simulated results of DPPPT dimers with an optimized intermolecular distance of 5.8 Å (Figure 2e and Figure S5b, Supporting Information). The intermolecular distance experimentally analyzed from the SAED pattern was ≈4.3 Å (Figure S6b, Supporting Information). This discrepancy between the simulated and experimental results might be attributable to differences in the medium solvent conditions (the simulations were performed under vacuum conditions). The discrepancy therefore suggests that the alkyl side chains of the DPPPT strongly influenced the self‐assembly process in toluene, unlike that in chloroform, where π–π interaction was dominant. Such molecular interactions might hinder the twisted face‐to‐face stacking of the DPP conjugated backbone, leading to a substantial reduction in the CD responses.

Figure 2c shows the most distinct feature of coiled coil‐like nanohelix architectures; the formation of intertwined nanofibers upon gelation with the DMF. A large *g*_abs_ value of 0.05 was attained as a result of the π–π interactions with high degrees of helical order of the flat DPP conjugated backbone (Figure 2f). We confirmed that this CD response is not due to macroscopic anisotropies, such as birefringence or linear dichroism. Negligible differences in the CD spectra were observed when the sample was viewed from the front and back or when the azimuthal angle of the sample was rotated around the optical axis of the incident light (Figure S7, Supporting Information). An SEM image of the DPPPT in DMF before gelation (i.e., at RT) shows a featureless morphology (Figure S8, Supporting Information). However, at the gelation temperature of 153 °C, the occurrence of hydrogen bonding between the amide groups of DPPPT and DMF promotes the self‐assembly of DPPPTs. The Fourier transform infrared (FT‐IR) spectra of the DMF‐gelated DPPPT films show absorption in the range 1700–1750 cm^−1^, indicative of a hydrogen‐bonded C═O band,^[^ ^49^, ^50^ ^]^ this band not observed in the spectra of the gels formed using other solvents (i.e., chloroform and toluene) (Figure S9, Supporting Information). The hydrogen bonding between the amide groups in neighboring DPPPT causes the steric hindrance at the interface between DPP core and bulky alkyl side chains, that facilitates twist stacking of DPP‐conjugated cores to maintain π–π interactions, ultimately resulting in the formation of helical nanofibers (Figure 2i). These nanofibers are eventually entangled with each other to compensate for the unfavorable contacts between conjugated backbone and DMF, forming a stable coiled coil‐like superhelix. Because of hydrogen bonding to the C═O covalently bonded to the chiral center of glutamic acid, the molecular chirality of (*R*)‐DPPPT and (*S*)‐DPPPT could be preserved in the nanohelix. The strong CD signals of the nanohelix were retained in the thin film prepared by spray‐coating the dispersion onto a substrate heated to 100 °C due to such strong intermolecular interactions (Figure S10, Supporting Information). In addition, it was confirmed that the CD response of the DPPPT thin film showed almost no change after storing the sample in the petridish under ambient condition for one year (Figure S11, Supporting Information). These results indicate that the DPPPT nanohelix is structurally and optically stable under certain heat and air exposures.

**Figure 3:**
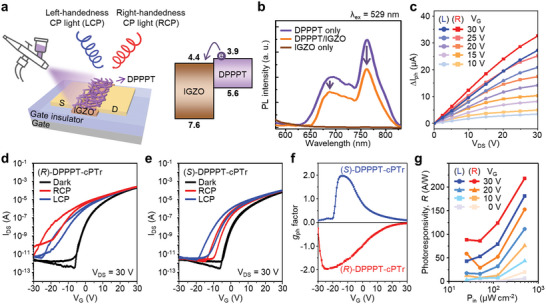
a) Schematic showing the heterojunction phototransistor consisting of DPPPT nanohelices deposited onto IGZO transistor, where the channel length and channel width are 50 and 1000 µm, respectively. The flat energy‐band diagram formed between DPPPT and IGZO, which facilitates the interfacial electron transfer from the LUMO of DPPPT to the conduction band of IGZO. b) PL spectra of the DPPPT (violet line), DPPPT/IGZO (orange line), and IGZO (brown line) on a quartz substrate. The laser pulse at a wavelength of 529 nm was used for photoexcitation. c) The photocurrents (∆*I*_ph_ = *I*_ph_ − *I*_dark_) of (*R*)‐DPPPT‐cPTr (10 ≤ *V*_G_ ≤ 30 V, *V*_G_ step = 5 V) upon irradiation with LCP (blue line) and RCP (red line) light (*λ* = 520 nm, *P*_in_ = 500 µW cm^−2^). d,e) Transfer curves of d) (*R*)‐DPPPT‐cPTr and e) (*S*)‐DPPPT‐cPTr under dark (black line) and CP light‐irradiated conditions (blue and red lines for LCP and RCP light, respectively). A *V*_SD_ of 30 V was applied to the drain electrode, and a CP light laser (*λ* = 520 nm, *P*_in_ = 500 µW cm^−2^) was used. f) Dissymmetry factor of the photocurrent (*g*_ph_) of DPPPT‐cPTr under the applied *V*_G_. Blue and red lines indicate the *g*_ph_ of (*S*)‐DPPPT‐cPTr and (*R*)‐DPPPT‐cPTr, respectively. g) Photoresponsivity of (*R*)‐DPPPT‐cPTr under irradiation with CP light with different light intensities. Gate biases of 0, 10, 20, and 30 V were applied.

To demonstrate high‐performance chiroptical photodetection, we designed a heterojunction phototransistor consisting of a DPPPT nanohelix deposited onto an IGZO field‐effect transistor with excellent charge‐transport behavior (**Figure** **3**a, left). The highest occupied molecular orbital (HOMO) and lowest unoccupied molecular orbital (LUMO) energy levels of DPPPT were estimated to be −5.6 and −3.9 eV, respectively, on the basis of photoemission spectroscopy (PES) and UV–vis absorption spectroscopy measurements (Figure S12, Supporting Information). Comparing the respective absorption spectra of DPPPT, IGZO, and DPPPT‐deposited on IGZO (DPPPT/IGZO), it was confirmed that no new electronic transition was observed after the DPPPT/IGZO heterojunction was formed (Figure S13, Supporting Information). Type‐II band alignment occurred between the DPPPT and IGZO, facilitating interfacial electron transfer from the LUMO level of DPPPT to the conduction band of IGZO (Figure 3a, right). The feasibility of interfacial charge transfer was confirmed by photoluminescence (PL) spectroscopic analysis. As shown in Figure 3b, when DPPPT was deposited onto the IGZO surface, the 30% of PL emissions at 690 and 763 nm induced by photoexcitation of DPPPT (*λ*_ex_ = 529 nm) were quenched compared with those of pristine DPPPT deposited at a same thickness onto a quartz substrate. The relatively low degree of PL quenching might be attributed to the fact that the charge transfer from the electron‐rich π‐conjugated backbone to IGZO can be hindered by the long alkyl chains surrounding the superhelix structure.

The CP light‐sensing characteristics of chiroptical heterojunction phototransistors were evaluated under irradiation with CP light with different rotation orientations (Figure 3c–e and Figure S14, Supporting Information). The demonstrated chiroptical phototransistors are referred to as (*R*)‐DPPPT‐cPTr and (*S*)‐DPPPT‐cPTr according to the handedness of the (*R*)‐DPPPT and (*S*)‐DPPPT nanohelices, respectively. The top side of the device was illuminated with monochromatic light sources with a wavelength of 520 nm and various optical powers. The output curves of DPPPT‐cPTr show chiroptical activities in response to irradiation with left circularly polarized (LCP) light and right circularly polarized (RCP) light (Figure 3c and Figure S14, Supporting Information). Parameter Δ*I*_ph_ represents the difference between the photocurrent generated by the illumination with CP light and the dark current (Δ *I*_ph_ = *I*_ph_  − *I*_dark_). The output curves of (*R*)‐DPPPT‐cPTr show that the RCP light illumination generated 1.2 times greater photocurrent compared with the LCP light illumination. The transfer characteristics show the corresponding CP light‐polarization‐dependent photocurrent generation due to the chirality of DPPPT nanohelices (Figures 3d and 3e). The *I*_DS_ hysteresis during the gate‐bias sweep and the negative shift of the threshold voltage (*V*_th_) of the DPPPT‐cPTr under illumination with CP light are attributed to the photo‐gating effect, where the photogenerated hole carriers are accumulated at the heterojunction interface.^[^ ^51^, ^52^, ^53^ ^]^ This light‐induced hole trapping at the heterojunction can lead to PPC, enabling the realization of a synaptic photodetector.^[^ ^33^, ^35^ ^]^ Details of the synaptic functionalities of the DPPPT‐cPTr are described below. The differences observed in photodetection performance between (*R*)‐DPPPT‐cPTr and (*S*)‐DPPPT‐cPTr might be due to the inevitable trial variations in the self‐assembly process. Previously reported circularly polarized light detectors based on chiral conjugated molecules have also exhibited performance differences between enantiomers.^[^ ^11^, ^54^, ^55^, ^56^ ^]^ Notably, the pristine IGZO transistor showed a negligible photoresponse under the same illumination conditions (Figure S15, Supporting Information), confirming that the significant distinction in the photocurrents for LCP and RCP light is due to the chiroptical property of the DPPPT nanohelix.

To quantitatively evaluate the CP light detection selectivity of DPPPT‐cPTr, we calculated the dissymmetry factor of the photocurrent (*g*_ph_) for both (*R*)‐DPPPT‐cPTr and (*S*)‐DPPPT‐cPTr (Figure 3f, bottom and top, respectively). The expression for extracting *g*_ph_ from the photoinduced transfer curves is

(1) $$g_{\mathrm{ph}}=\;2\frac{\left(I_{\mathrm{ph}}^{L}-\;I_{\mathrm{ph}}^{R}\right)}{\left(I_{\mathrm{ph}}^{L}+\;I_{\mathrm{ph}}^{R}\right)}$$

where $I_{\mathrm{ph}}^{L}$ and $I_{\mathrm{ph}}^{R}$ are the photocurrents induced by LCP and RCP light, respectively. Remarkably, the *g*_ph_ of for (*S*)‐DPPPT‐cPTr and (*R*)‐DPPPT‐cPTr reached +1.97 and −1.97, respectively (Figure 3f). This *g*‐factor of DPPPT‐cPTr is superior to those reported for previously developed CP light photodetectors based on inorganic materials (e.g., *g* ≈ 1.1),^[^ ^22^ ^]^ perovskites (e.g., *g* ≈ 1.8),^[^ ^57^, ^58^ ^]^ organic crystals (e.g., *g* ≈ 0.1),^[^ ^56^ ^]^ organic thin films (e.g., *g* ≈ 1.9),^[^ ^29^, ^54^, ^59^, ^60^ ^]^ or plasmonic nanoparticles (e.g., *g* ≈ 0.55).^[^ ^24^ ^]^

To evaluate the photodetection performance of the device, we analyzed the photoresponsivity (*R*), photocurrent/dark current ratio (*P*), specific detectivity (D*), and external quantum efficiency (*EQE*). Details of the characterization and calculation methods are described in the Section 4. As shown in Figure 3g, the *R* values of (*R*)‐DPPPT‐cPTr for RCP light at various light intensities were higher than those for LCP light. Conversely, for the (*S*)‐DPPPT‐cPTr, the opposite result was observed (Figure S16, Supporting Information). The maximum *R* value exceeded 218 A W^−1^ at a gate voltage of 30 V because of the bias‐driven amplification effect of the phototransistor. Notably, the *R* value of the DPPPT‐cPTr tend to increase with increasing excitation intensity because of IGZO's excellent charge‐carrier mobility. This behavior differs from that of previously reported photodiodes or phototransistors based on chiral conjugated molecules, which exhibited a decrease in *R* with increasing optical power because of an increase in charge‐carrier recombination and limited charge‐carrier mobility.^[^ ^61^ ^]^ The (*R*)‐DPPPT‐cPTr and (*S*)‐DPPPT‐cPTr exhibited D* values of 3.33 × 10^14^ and 2.52 × 10^13^ Jones (1 Jones = 1 cm Hz^1/2^ W^−1^) under preferential CP light illumination of 500 µW cm^−2^, respectively (Figure S17a,d, Supporting Information). Moreover, the *P* values were as high as 3.63 × 10^6^ for (*R*)‐DPPPT‐cPTr and 2.85 × 10^5^ for (*S*)‐DPPPT‐cPTr at a gate bias of −6 V, indicating that direct CP light detection with a large signal‐to‐noise ratio was achieved (Figure S17b,e, Supporting Information). The *EQE* of (*R*)‐DPPPT‐cPTr and (*S*)‐DPPPT‐cPTr, defined as the ratio of the number of generated carriers that enhance the drain current to the number of photons incident onto the channel area were 518% and 351%, respectively (Figure S17c,f, Supporting Information). These chiroptical photodetection parameters for DPPPT‐cPTr are compared in Table S1, Supporting Information to those of previously reported chiroptical photodetectors based on various nanomaterials. The results indicate that, by combining the strong and stable chiroptical activities of the DPPPT nanohelix and the excellent charge transport of an IGZO field‐effect transistor, we successfully fabricated a chiroptical phototransistor that demonstrates superior performance compared with conventional chiroptical photodetectors.

**Figure 4:**
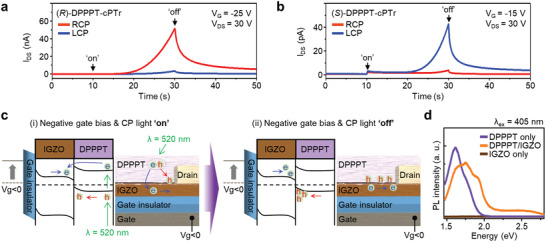
a,b) Time‐dependent photocurrent generation and decay characteristics of a) (*R*)‐DPPPT‐cPTr and b) (*S*)‐DPPPT‐cPTr under continuous illumination with CP light for 20 s (*λ* = 520 nm, *P*_in_ = 500 µW cm^−2^). At *V*_SD_ = 30 V, a gate bias of −25 and −15 V was applied to the gate of the (*R*)‐DPPPT‐cPTr and (*S*)‐DPPPT‐cPTr device, respectively (blue and red lines for LCP and RCP light, respectively). c) Energy‐band diagram of a DPPPT/IGZO heterojunction and a schematic of the DPPPT‐cPTr device, showing the photocurrent generation mechanism in DPPPT‐cPTr. d) PL spectra of the DPPPT (violet line), DPPPT/IGZO (orange line), and IGZO (brown line) on a quartz substrate. Laser pulses with a wavelength of 405 nm were used for photoexcitation.

Interestingly, the DPPPT‐cPTr device exhibited photonic synapse‐like, time‐dependent photocurrent generation and PPC under illumination with CP light. As shown in **Figure** **4**a, the *I*_DS_ of (*R*)‐DPPPT‐cPTr started to abruptly increase under irradiation with RCP light for 20 s when a gate bias of −25 V was applied, whereas irradiation with LCP light led to a negligible photocurrent. The opposite trend was observed for (*S*)‐DPPPT‐cPTr (Figure 4b). As a possible mechanism of such time‐dependent photocurrent behavior of the DPPPT‐cPTr, we propose a synergetic effect between i) the interfacial charge trapping at the DPPPT/IGZO heterojunction (Figures 4c) and ii) oxygen vacancy ionization of IGZO induced by DPPPT deposition. From the viewpoint of the photocurrent generation process under CP light illumination, the excitons (i.e., electron–hole pairs) photogenerated by selective light absorption in the chiral DPPPTs are dissociated into free electron and hole carriers. The photogenerated electrons in the LUMO of DPPPT can then be transferred to the conduction band of IGZO, and transport of these delocalized electrons through the IGZO channel increases *I*_DS_, referred to as the photocurrent. Meanwhile, the photogenerated holes energetically tend to move toward the IGZO and can be localized at the trap sites near the DPPPT/IGZO interface. The interfacial hole trapping can be retained even after the light source is turned off, leading to PPC. Recombination (or de‐trapping) of the trapped holes does not favorably occur at the negative gate bias because of the deficiency of the delocalized electrons induced by the limited electron injection and fast electron transport in the IGZO. Therefore, during prolonged irradiation, more photogenerated holes can accumulate at the IGZO/DPPPT interface, leading to a more negative shift of the *V*_th_ by the photogating effect. When the *V*_th_ is sufficiently shifted to induce charge injection at the source by continuous irradiation, the *I*_DS_ will start to increase, leading to an abrupt increase of the photocurrent.

In addition, such induced hole trapping at the DPPPT/IGZO heterojunction can contribute to the oxygen vacancy ionization of the IGZO. According to the literature, oxygen vacancy ionization ($V_{O}^{0}\;\to \;V_{O}^{2+}+2e^{-}$) in IGZO thin films can be induced under visible‐light illumination.^[^ ^62^ ^]^ The electron‐deficient hole traps formed at the DPPPT/IGZO interface might facilitate the ionization reaction of the oxygen vacancies. According to the density of state (DOS) model for IGZO, the excited electrons in the IGZO can be trapped by a positively charged oxygen vacancy ($V_{O}^{2+}$) formed inside the bandgap and then recombined with the thermalized positive holes trapped deep in the valence band tail states, which has been experimentally observed to result in 1.82 eV PL emission.^[^ ^63^, ^64^ ^]^ In fact, when the DPPPT/IGZO film was excited with high‐energy optical light (3.06 eV) of 405 nm wavelength, this characteristic PL emission band was observed at ≈1.75 eV (Figure 4d). Such deep‐trap‐driven PL emission was observed only for the DPPPT/IGZO heterojunction, not for the pristine IGZO and DPPPT films, verifying that a DPPPT/IGZO heterojunction induces oxygen vacancy ionization and the formation of deep traps in the bandgap of the IGZO. The experiments also confirmed that the PPC of the DPPPT‐cPTr was recovered when a positive gate bias (i.e., +30 V) was applied (Figure S18, Supporting Information). In addition, the threshold time at which a sudden photocurrent increase occurred could be tuned by modulating the gate bias (Figure S19, Supporting Information). These results imply that the interfacial hole trapping and formation of oxygen vacancy deep traps occur at the chiral DPPPT and IGZO heterojunction under irradiation, leading to a photonic synapse‐like, time‐dependent photomemory effect of the DPPPT‐cPTr by the CP light. Regarding to the stability of the device, it was confirmed that such synaptic photoresponses of DPPPT‐cPTr were reproducibly observed at the same device after storing it for one month in the vacuum (Figure S20, Supporting Information).

It should be mentioned that the low‐voltage operational DPPPT‐cPTr can be achieved by using a high‐k Al_2_O_3_ gate dielectric instead of 300 nm SiO_2_ gate dielectric. Figure S21a,b, Supporting Information exhibits the output and transfer characteristics of (*R*)‐DPPPT‐cPTr with 50 nm‐thick Al_2_O_3_ gate dielectric. The polarization‐dependent photoresponses consistent with the optoelectrical properties of (*R*)‐DPPPT‐cPTr operated at a high V_DS_ of 30 V was obtained at a low *V*_DS_ of 5 V. The low‐voltage operational (*R*)‐DPPPT‐cPTr also exhibited characteristic time‐dependent photocurrent generation behavior with polarization selectivity under CP light illumination. This demonstrates that the proposed DPPPT‐cPTr has potential for use in low‐power optoelectronic systems in the future.

**Figure 5:**
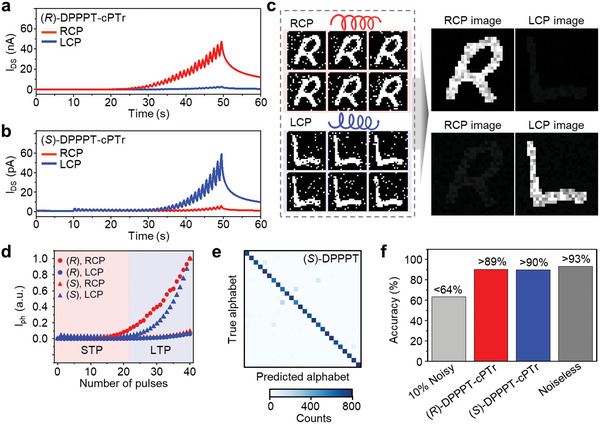
a,b) Time‐dependent photocurrent generation and decay characteristics of a) (*R*)‐DPPPT‐cPTr and b) (*S*)‐DPPPT‐cPTr in response to illumination by pulsed CP light (wavelength: 520 nm, *P*_in_ = 500 µW cm^−2^, duration: 0.5 s, frequency: 1 Hz). Blue and red lines indicate the photoresponses to LCP and RCP light, respectively. At *V*_SD_ = 30 V, a gate bias of −25 and −15 V was applied to the gate of the (*R*)‐DPPPT‐cPTr and (*S*)‐DPPPT‐cPTr device, respectively. c) Schematic showing the chiroptical imaging while reducing the noise, performed by (*R*)‐DPPPT‐cPTr (right top) and (*S*)‐DPPPT‐cPTr (right bottom). DPPPT‐cPTr can derive noise‐reduced images from the sequential illumination of noisy alphabet images while distinguishing the CP light orientation. d) Normalized photocurrent of (*R*)‐DPPPT‐cPTr (dots) and (*S*)‐DPPPT‐cPTr (triangles) under irradiation with LCP (blue) and RCP (red) light. The red and blue shaded areas represent the short‐term firing (STP) and long‐term firing (LTP) regions, respectively, depending on the number of light pulses. e) Convolution matrix for the simulated images that can be obtained using (*S*)‐DPPPPT‐cPTr. f) Recognition rate of noisy images with 10% flipped pixels (light‐gray), simulated images that can be obtained using (*R*)‐DPPPPT‐cPTr (red) and (*S*)‐DPPPT‐cPTr (blue), and noiseless images (dark‐gray).

On the basis of the synaptic photoresponse of DPPPT‐cPTr, we demonstrated selective detection of LCP and RCP light while filtering the background noise (e.g., salt‐and‐pepper noise).^[^ ^65^ ^]^ We first investigated the photocurrent generation characteristics of DPPPT‐cPTr in response to repeated CP light pulses (i.e., light pulses with a wavelength of 520 nm, duration of 0.5 s, and frequency of 1 Hz) (**Figures** **5**a,b). The applied gate bias was fixed at −25 and −15 V for (*R*)‐DPPPT‐cPTr and (*S*)‐DPPPT‐cPTr, respectively, where the maximum *g*_ph_ values were observed. Notably, negligible photocurrent was generated upon irradiation of the (*S*)‐DPPPT‐cPTr with RCP pulses. In addition, nearly zero photocurrent was observed under irradiation with as many as 20 LCP pulses, indicating that such inputs can be filtered out by (*S*)‐DPPPT‐cPTr. However, when more than 20 LCP pulses were used, a large photocurrent was generated and the photocurrent was nonlinearly dependent on the number of CP light pulses. These results suggest that only the frequent LCP data that might be meaningful can be extracted. A similar tendency was observed for (*R*)‐DPPPT‐cPTr (Figure 5a). Therefore, DPPPT‐cPTr can emphasize the frequent CP light of the target rotation orientation while filtering the infrequent CP light of the target rotation orientation and opposite‐handed CP light. These attributes of DPPPT‐cPTr can be used to acquire a noise‐reduced CP light image of the target rotating orientation from the sequential illumination of noisy images through a single readout operation.^[^ ^29^, ^32^, ^33^, ^36^ ^]^ The noise‐reduced images can be used to achieve highly accurate image recognition.^[^ ^35^ ^]^

To demonstrate the effectiveness of DPPPT‐cPTr in noise‐reduced chiroptical imaging, the recognition of images of handwritten alphabet characters was carried out using a convolutional neural network (CNN). Initially, we imported 20,800 test images from the Extended Modified National Institute of Standards and Technology (EMNIST) dataset and digitized the pixel intensities to prepare noiseless binary images of handwritten alphabets. The noisy alphabet images were then generated by flipping 10% of the pixels of the noiseless binary images (Figure 5c, left). We subsequently simulated the images that can be obtained from sequential illumination of 20 noisy alphabet images to (*R*)‐DPPPT‐cPTr and (*S*)‐DPPPT‐cPTr using their respective empirical parameters (Figure 5c, right). These empirical parameters were derived from the normalized photocurrent values generated depending on the number of CP light pulses (Figure 5d). Using the number of CP light pulses incident on each pixel of the 28 × 28 DPPPT‐cPTr array, we calculated and mapped the normalized photocurrent values, obtaining the simulated images. As shown in Figure 5c, (*R*)‐DPPPT‐cPTr selectively detected only RCP images while neglecting LCP images, and vice versa for (*S*)‐DPPPT‐cPTr. The background noise shown in the noisy input images was not observed in the detected LCP images, indicating successful noise reduction in chiroptical imaging. The effectiveness of noise‐reduced chiroptical imaging by DPPPT‐cPTr was evaluated by comparing the recognition rates of the simulated images as well as the noiseless images and the noisy images using the CNN trained with 124,800 noiseless training images for 5 epochs. Figure 5e shows the confusion matrix indicating the classification results of the simulated alphabet images for (*S*)‐DPPPT‐cPTr. Although the recognition rate of noiseless images was 93.2% (dark‐gray bar in Figure 5f), the recognition of noisy images with 10% flipped pixels decreased to <63.4% (light‐gray bar in Figure 5f). By contrast, the recognition rate of the simulated images for (*R*)‐DPPPT‐cPTr and (*S*)‐DPPPT‐cPTr reached ≈89.7% and ≈90.1%, respectively (red and blue bars in Figure 5f, respectively), which are comparable to the recognition rate of the noiseless images (≈93.2%). This improvement is attributed to the reduction of the background noise by DPPPT‐cPTr.^[^ ^35^ ^]^ These results suggest that DPPPT‐cPTr has strong potential for use in high‐performance chiroptical imaging based on synaptic functionality. However, by applying the negative gate bias, highly sensitive chiroptical imaging with a large signal‐to‐noise can be achieved owing to large *g*_ph_ and *P*. Nevertheless, drain current values are relatively low in those regions, necessitating the use of a high‐resolution analog‐to‐digital converter to measure the tiny current.

## 3 Conclusion

High‐performance chiroptical synaptic phototransistors were successfully demonstrated using a heterojunction formed between a self‐assembled nanohelix of π‐conjugated molecules and an IGZO semiconductor. A novel DPP‐based chiral π‐conjugated molecule decorated with chiral glutamic acid capable of various noncovalent interactions for supramolecular self‐assembly was newly synthesized. The fundamental relation among intermolecular interactions, self‐assembled structure, and chiroptical properties was thoroughly investigated. In particular, the hydrogen‐bonding‐driven gelation of the chiral DPP molecule was found to result in a coiled coil‐like nanohelix with strong chiroptical absorption in the film state. On the basis of interfacial charge transfer from the nanohelix to the IGZO semiconductor under illumination with CP light, we developed a high‐performance chiroptical phototransistor that demonstrated excellent distinguishability of CP light with a high dissymmetry factor, as well as superior photoresponsivity and detectivity. In addition, the chiroptical phototransistor showed photonic synapse‐like, time‐dependent photocurrent generation and PPC, which was attributed to a synergistic effect of the interfacial hole trapping at the chiral nanohelix/IGZO heterojunction and the formation of positively charged oxygen vacancies in the IGZO. With the advantage of synaptic functionality, upon the irradiation of noisy images, neural‐network‐based image recognition of CP light with high accuracy was successfully demonstrated. We believe this study provides a promising strategy to utilize not only chiroptical π‐conjugated molecules but also their various supramolecular nanostructures in the development of high‐performance chiral optoelectronic devices.

## 4 Experimental Section

### Materials

*N*‐(tert‐Butoxycarbonyl)‐l‐glutamic acid (Boc‐l‐glutamic acid) and *N*‐(tert‐butoxycarbonyl)‐d‐glutamic acid (Boc‐d‐glutamic acid) were purchased from TCI, Japan. Dodecylamine, COMU, *N*,*N*‐diisopropylethylamine (DIEA), 4‐carboxylphenylboronic acid pinacol ester, trifluoroacetic acid, trioctylmethylammonium chloride (Aliquat 336), tris(dibenzylideneacetone)dipalladium(0) (Pd_2_(dba)_3_), tri(*o*‐tolyl)‐phosphine (P(*o*‐tol)_3_), and potassium carbonate (K_2_CO_3_) were purchased from Sigma‐Aldrich. 3,8‐Bis(5‐bromothiophene‐2‐yl)‐2,5‐bis(2‐octyldodecyl)pyrrolo[3,4,‐*c*]pyrrole‐1,4(2*H*,5*H*)‐dione (2Br‐DPP) was received from Solarmer Materials, China. The solvents used for gelation, such as chloroform, toluene, and DMF, were purchased from Sigma‐Aldrich and used without further purification.

### Synthesis of DPPPT

The DPPPT was synthesized via a COMU coupling reaction and a Suzuki–Miyaura coupling reaction under an Ar atmosphere. The detailed synthesis procedure is described in Method S1, Supporting Information.

### Materials Characterization

^1^H‐NMR spectra were recorded using a 400 MHz NMR spectrometer (Avance III, Bruker). MALDI‐TOF mass spectrometry was performed using a MALDI‐TOF mass spectrometer (SCIEX TOF/TOF 5800, SCIEX). The optical absorption and circular dichroism (CD) spectra of DPPPT solutions and films were acquired using a UV–vis–NIR spectrophotometer (Lambda 750, PerkinElmer) and a CD spectrophotometer (J‐815, JASCO), respectively. The morphology of the DPPPT films was investigated by SEM (Nova Nano SEM 200, FEI) and TEM (JEM‐1400, JEOL Co.) at an accelerating voltage of 120 kV, and the images were captured with a  side‐mounted 2k x 2k Veleta CCD camera (Olympus‐SiS, Munster, Germany). The crystal structures were determined using on the selected area electron diffraction (SAED) pattern obtained from the TEM. The ionization potentials of the DPPPT films were obtained using a photoelectron (PE) yield spectrometer (AC2, Riken Keiki). The attenuated total reflectance (ATR) transmittance spectra of the DPPPT films were acquired using an FT‐IR spectrometer (Spectrum 100, PerkinElmer). PL spectra were recorded using a PL spectrophotometer (FP‐6500, JASCO).

### Quantum Chemical Calculations of DPPPT

The simulated optical absorption results were obtained using the DFT calculations as implemented in the plane‐wave pseudopotential approach in the Vienna Ab initio Simulation Package (VASP^[^ ^66^ ^]^). The electron–core interaction was expressed by the projector augmented‐wave (PAW^[^ ^67^ ^]^) method, and the gradient‐corrected exchange‐correlation general gradient approximation (GGA) functional of Perdew–Burke–Ernzerhof (PBE^[^ ^68^ ^]^) was used for all the calculations. A kinetic energy cutoff of 500 eV was imposed for the plane‐wave basis set, and Monkhorst–Pack sampling was used for the Brillouin zone integration.

### Fabrication of DPPPT‐cPTr

To fabricate a bottom‐gate/top‐contact IGZO transistor, a heavily *p*‐doped Si wafer with a SiO_2_ dielectric layer (thickness ∼200 nm) was used as a substrate. IGZO (Ga_2_O_3_/In_2_O_3_/ZnO = 1:1:1 mol%) active layers (50 nm) were deposited using a radio‐frequency (RF) sputtering system set to 35 W and a working pressure of 1.6 mTorr of Ar gas (143 sccm) and O_2_ gas (7.1 sccm). The IGZO films were patterned by wet‐etching to define the channel (channel length (*L*) of 50 µm and channel width (W) of 1000 µm) and then annealed at 350 °C for 1 h under ambient conditions. The Au/Ti source/drain electrodes (50/25 nm, respectively) were deposited by direct‐current (DC) sputtering at 50 W for Au and 100 W for Ti with a working pressure of 5 mTorr of Ar gas (40 sccm) and were patterned by a conventional lift‐off process. Finally, the device was annealed at 160 °C under vacuum for 1 h for the IGZO oxygen reduction reaction. The DPPPT gel formed in DMF was spray‐coated onto the IGZO transistor while the device was annealed at 100 °C.

### Electrical Characterization

The electrical properties of the fabricated devices were characterized under dark and light‐illuminated conditions using Keithley 4200 and Agilent 4155B semiconductor characterization system (SCS) parameter analyzers. Laser diodes emitting light with a wavelength of 520 nm (L520P50, Thorlabs) were used to illuminate the devices. CP light was obtained by combining a linear polarizer (10GT04, Newport) with a quarter‐wave plate (SAQWP05M‐700, Thorlabs). The on/off modulation of incident CP light was controlled by a mechanical optical shutter (SH1, Thorlabs). Before the characterization, the intensities of the RCP and LCP light (*P*_in_) were measured using a standard Si‐based photodetector (DET10A, Thorlabs).

### Calculation of Photodetection Parameters

The *R*, *P*, EQE, and D* values were directly extracted from photoinduced transfer curves (*λ*  = 520 nm, *P*_in_ = 500 µW cm^−2^) in Figure 3d,e using the following equation:

(2) $$\mathrm{Photoresponsivity}\;\;\left(R\right)=\;\frac{\left(I_{\mathrm{light}}-I_{\mathrm{dark}}\right)}{P_{\mathrm{in}}\cdot A}$$

(3) $$\mathrm{External}\;\mathrm{quantum}\;\mathrm{efficiency}\;\;\left(\mathrm{EQE}\right)=\;\frac{(I_{\mathrm{light}}-I_{\mathrm{dark}})\cdot hc}{P_{\mathrm{in}}\cdot q\cdot A\cdot \lambda }$$

(4) $$\mathrm{Photocurrent}/\mathrm{dark}\;\mathrm{ratio}\;\;\left(P\right)=\;\frac{\left(I_{\mathrm{light}}-I_{\mathrm{dark}}\right)}{I_{\mathrm{dark}}}$$

(5) $$\mathrm{Specific}\;\mathrm{detectivity}\;\;\left(D^{*}\right)=\;\frac{R}{\sqrt{\left(2\cdot q\cdot I_{\mathrm{dark}}/A\right)}}$$

where *I*_light_ is the drain current under CP light illumination, *I*_dark_ is the drain current in the dark, *P*_in_ is the incident light intensity measured by a power meter, *A* is the channel area, *h* is Planck's constant, *c* is the speed of light, *q* is the fundamental unit of charge, and λ is the wavelength of incident light.

### Simulation of Convolutional‐Neural‐Network‐Based Image Recognition

The handwritten alphabet images were imported from the EMNIST dataset, which consisted of 124,800 training images and 20,800 test images. The intensities of test images, ranging from 0 to 255, were digitized to either 1 or 0 on the basis of whether the intensity was greater or less than the threshold value of 128, respectively. This process was used to prepare noiseless binary images for testing. Then, 20 noisy images were generated by randomly flipping the 10% pixels of the noiseless binary images for testing. Using the normalized photocurrent values of (*S*)‐DPPPT‐cPTr and (*R*)‐DPPPT‐cPTr depending on the number of incident CP light pulses (Figure 5d), simulated images acquired upon sequential illumination of 20 noisy images were obtained for each device.

The CNN model for classifying the alphabet images was implemented using Python with the Tensorflow library. The architecture of the CNN model consisted of two convolutional layers, each of which included 32 and 64 filters, respectively, 3 × 3 kernels, and the ReLU activation function. Max pooling was then applied with a 2 × 2 pool size. To reduce overfitting, dropout layers with rates of 25% and 50% were added. The CNN model was trained for 5 epochs using the 124,800 noiseless handwritten alphabet. Adaptive moment estimation (ADAM) was used as an optimizer, and sparse_categorical_corssentropy was used as a loss function. The recognition rates of noiseless test images, noisy images, and simulated images for (*S*)‐DPPPT‐cPTr and (*R*)‐DPPPT‐cPTr were evaluated using the trained CNN.

## Conflict of Interest

The authors declare no conflict of interest.

## Supporting information

Supporting Information

## Data Availability

The data that support the findings of this study are available from the corresponding author upon reasonable request.
