# Supplementary material for: Chiroptical Synaptic Heterojunction Phototransistors Based on Self‐Assembled Nanohelix of π‐Conjugated Molecules for Direct Noise‐Reduced Detection of Circularly Polarized Light
Source: Adv Sci (Weinh). 2023 Jul 27;10(27):2304039. doi: 10.1002/advs.202304039 (PMC10520648; doi:10.1002/advs.202304039)
Supplement: Supplementary file 1 — Supporting Information [file ADVS-10-2304039-s001.pdf]

ADVANCED SCIENCE
Open Access

Supporting Information

for *Adv. Sci.*, DOI 10.1002/advs.202304039

Chiroptical Synaptic Heterojunction Phototransistors Based on Self-Assembled Nanohelix of π-Conjugated Molecules for Direct Noise-Reduced Detection of Circularly Polarized Light

Hanna Lee, Jun Ho Hwang, Seung Ho Song, Hyemi Han, Seo-Jung Han, Bong Lim Suh, Kahyun Hur, Jihoon Kyhm, Jongtae Ahn, Jeong Ho Cho, Do Kyung Hwang, Eunji Lee, Changsoon Choi* and Jung Ah Lim**

Supporting Information

Chiroptical synaptic heterojunction phototransistors based on self-assembled nanohelix of π-conjugated molecules for direct noise-reduced detection of circularly polarized light

Hanna Lee, Jun Ho Hwang, Seung Ho Song, Hyemi Han, Seo-Jung Han, Bong Lim Suh, Kahyun Hur, Jihoon Kyhm, Jongtae Ahn, Jeong Ho Cho, Do Kyung Hwang, Eunji Lee, Changsoon Choi , Jung Ah Lim

H. Lee, S. H. Song, Dr. H. Han, Dr. J. Ahn, Dr. D. K. Hwang, Dr. C. Choi, Dr. J. A. Lim
Center for Opto-Electronic Materials and Devices
Korea Institute of Science and Technology
Seoul 02792, Republic of Korea
E-mail: cschoi91@kist.re.kr, jalim@kist.re.kr

H. Lee, Prof. J. H. Cho
Department of Chemical and Biomolecular Engineering
Yonsei University
Seoul 03722, Republic of Korea

J. H. Hwang, Prof. E. Lee
School of Materials Science and Engineering
Gwangju Institute of Science and Technology
Gwangju 61005, Republic of Korea
E-mail: eunjilee@gist.ac.kr

Dr. S. -J. Han
Chemical & Biological Integrative Research Center
Korea Institute of Science and Technology
Seoul 02792, Republic of Korea

Dr. S. -J. Han
Division of Bio-Medical Science & Technology
KIST School
University of Science and Technology of Korea
Seoul 02792, Republic of Korea

Dr. B. L. Suh, Dr. K. Hur
Extreme Materials Research Center
Korea Institute of Science and Technology
Seoul 02792, Republic of Korea

Dr. J. Kyhm
Technology Support Center
Korea Institute of Science and Technology
Seoul 02792, Republic of Korea

Dr. D. K. Hwang
KU-KIST Graduate School of Converging Science and Technology
Korea University
Seoul 02841, Republic of Korea

Dr. D. K. Hwang, Dr. J. A. Lim
Division of Nano and Information Technology
KIST School
University of Science and Technology
Seoul 02792, Republic of Korea

Method S1. Synthesis of DPPPT

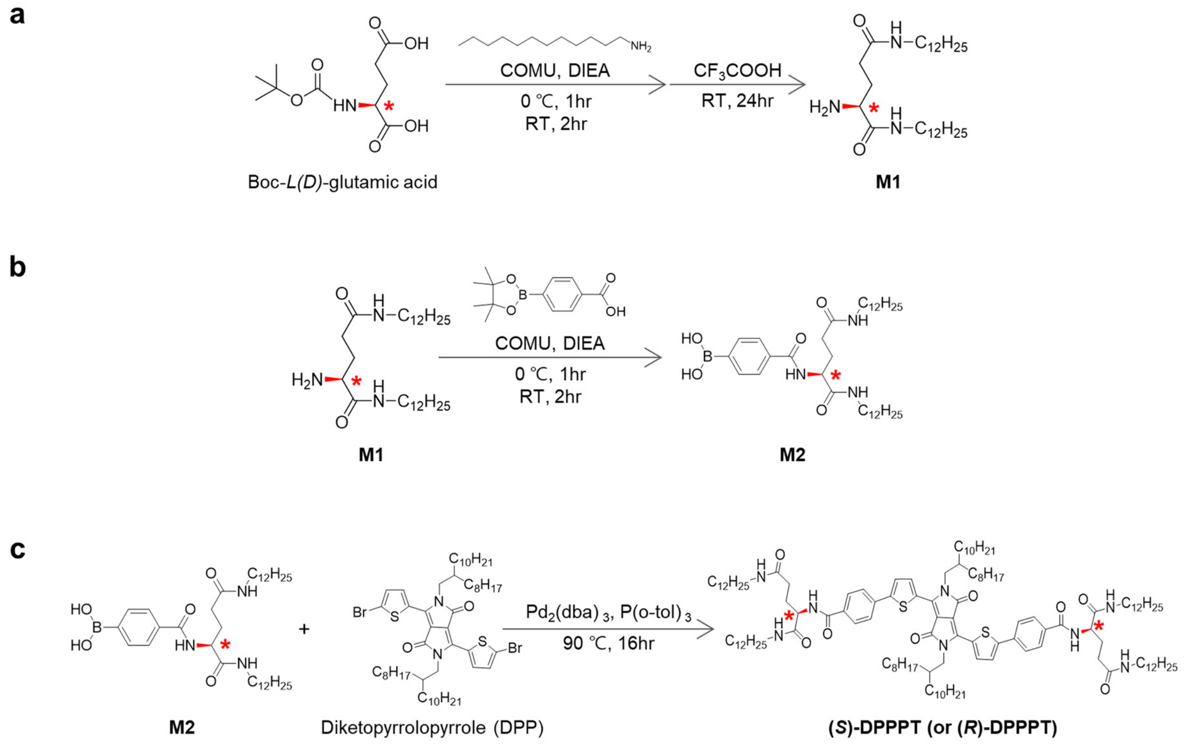

Figure S1. Synthesis scheme for the DPPPT via a COMU coupling reaction and a Suzuki–Miyaura coupling reaction.

Synthesis of M1 (Figure S1a): In a flask, a mixture of Boc-*L*-glutamic acid (or Boc-*D*-glutamic acid) (1.11 g) and dodecylamine in dichloromethane was stirred at 0 °C. COMU and DIEA were then added. The reaction mixture was stirred at 0 °C for 1 h and then at room temperature (RT) for 2 h under an Ar atmosphere. After reaction, the mixture was diluted with ethyl acetate and extracted with saturated $NaHCO_3$, saturated NaCl, and deionized (DI) H_2O. The combined organic phase was dried over $MgSO_4$. After the solvents were removed, the residues were dissolved thoroughly in THF and the resultant solutions were poured into a stirring 10 wt% Na_2CO_3 aqueous solution. The collected precipitate was filtered and dried under vacuum to obtain the crude product. The obtained crude product and trifluoroacetic acid were dissolved in dichloromethane, and the resultant mixture was stirred at room temperature for 24 h. After all of the solvents were removed, the crude product was dissolved thoroughly in ethanol and poured into a stirring 10 wt% Na_2CO_3 aqueous solution. Recrystallization from methanol obtained the white pure product (1.07 g, yield: 50%). ^{1}H-NMR (400 MHz, chloroform-*d*, δ): 7.35 (s, 1H), 6.11 (s, 1H), 3.43 (t, J = 6.6 Hz, 1H), 3.23 (m, 4H), 2.33 (m, 2H), 1.95 (m, 2H), 1.65 (s, 2H), 1.49 (m, 4H), 1.26 (m, 36H), 0.88 (t, J = 6.5, 6H)

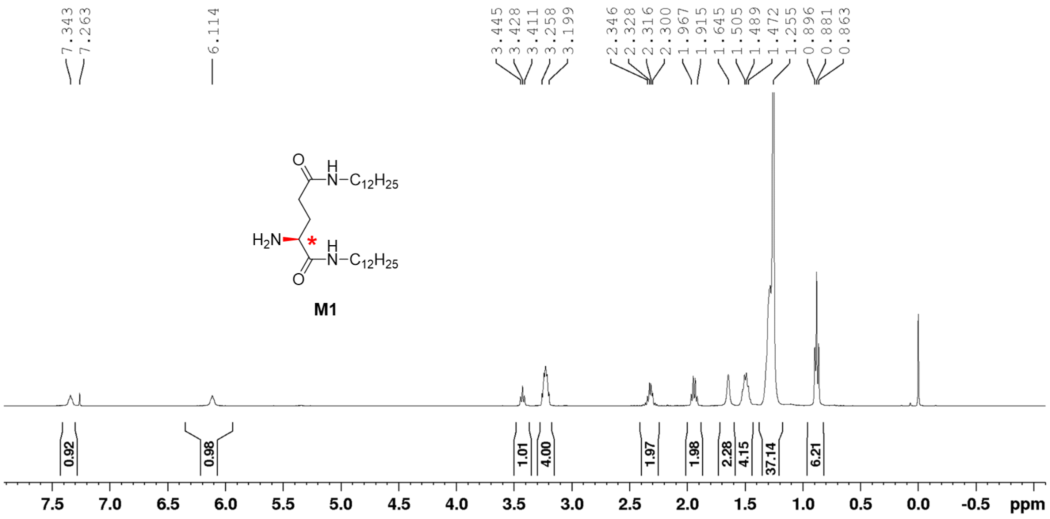

Synthesis of M2 (Figure S1b): M1, 4-carboxylphenylboronic acid pinacol ester (0.24 g), and dichloromethane as a solvent were added to a flask, and the resultant mixture was stirred at 0 °C. COMU and DIEA were then added. The reaction mixture was stirred at 0 °C for 1 h and then at RT for 2 h under an Ar atmosphere. After reaction, the mixture was diluted with ethyl acetate and extracted with saturated $NaHCO_3$, saturated NaCl, and DI H_2O. The combined organic phase was dried over $MgSO_4$. After removal of solvents, the residues were dissolved thoroughly in ethanol and then subsequently poured into stirring 10 wt% Na_2CO_3 aqueous solution. The collected precipitate was filtered and dried under vacuum to obtain the crude product. Recrystallization from DI H_2O/methanol (60 % v/v) gave the white pure product (0.54 g, yield: 88%). ^{1}H-NMR (400 MHz, chloroform-*d*, *δ*): 8.42 (d, *J* = 7.7 Hz, 1H), 8.18 (m, 1H), 7.84 (m, 5H), 4.33 (m, 1H), 3.03 (m, 4H), 2.02 (m, 4H), 1.30 (m, 40H), 0.85 (m, 6H).

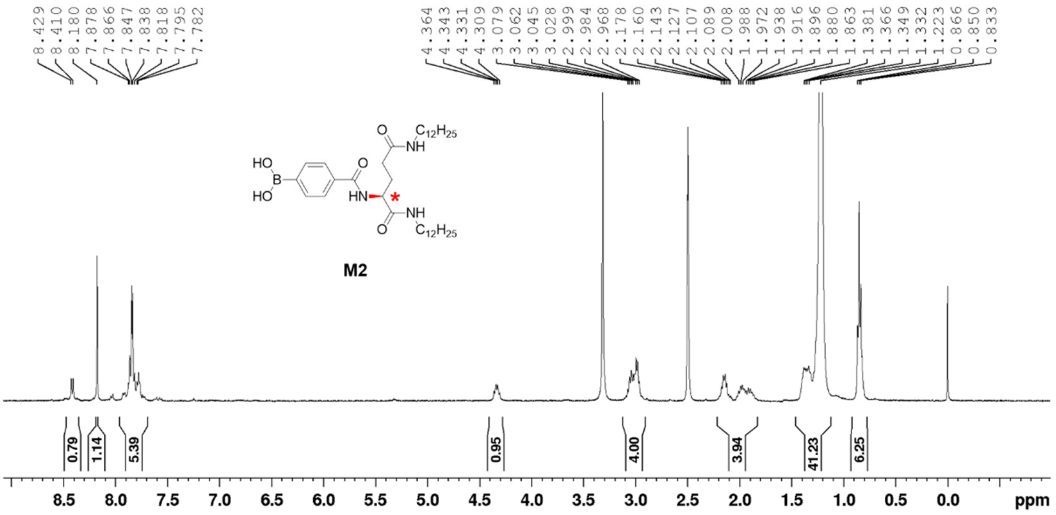

Synthesis of DPPPT (Figure S1c): 2Br-DPP (0.29 g), M2 (0.40 g), anhydrous K_2CO_3 (316 mg), and deoxygenated toluene were added to a flask under N_2, and the resultant mixture was stirred at room temperature until it became homogeneous (~10 min). $Pd_2(dba)_3$ (5.24 mg) and P(*o*-tol)$_3$ (6.97 mg) were then added to the mixture under N_2. DI H_2O and 100 μL of Aliquat® 336 were subsequently injected into the mixture. The reaction mixture was stirred at 90 °C for 16 h under an Ar atmosphere. After cooling to room temperature, the reaction mixture was precipitated into methanol. The precipitates were filtrated and dried under vacuum to afford the desired product as a purple solid. Recrystallization from chloroform/methanol (5 % v/v) gave the white pure product (0.49 g, yield: 84 %) ^{1}H-NMR (400 MHz, chloroform-*d*, *δ*): 8.94 (m, 2H), 8.25 (m, 2H), 7.97 (m, 4H), 7.74 (m, 4H), 7.54 (m, 2H), 6.92 (m, 2H), 5.85 (s, 2H), 4.55 (m, 2H), 4.06 (m, 4H), 3.27 (m, 8H), 2.30 (m, 8H), 1.56 (m, 10H), 1.24 (m, 128H), 0.86 (m, 32H).

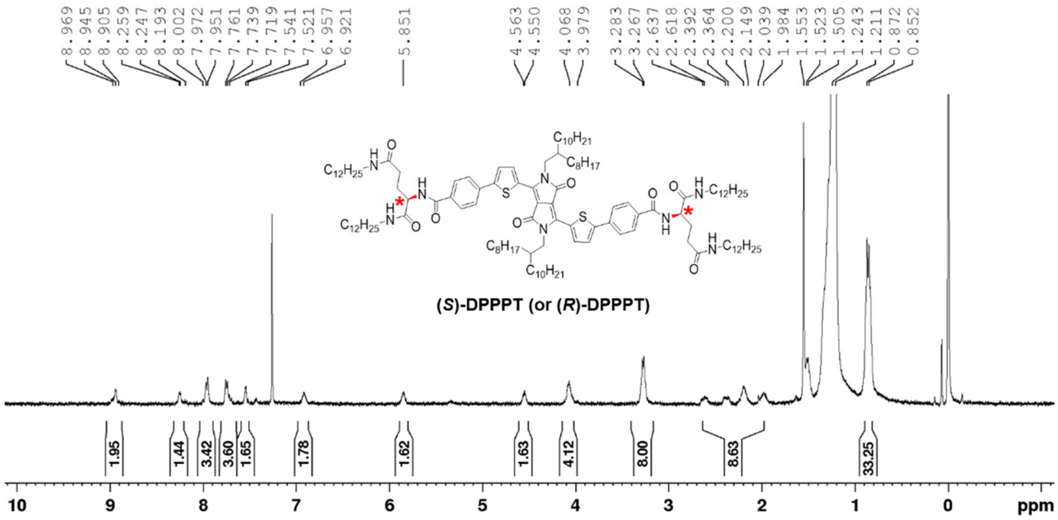

MALDI-TOF: *m/z* calculated for $C_{126}H_{210}N_8O_8S_2$ 2028.57, found 2029.49.

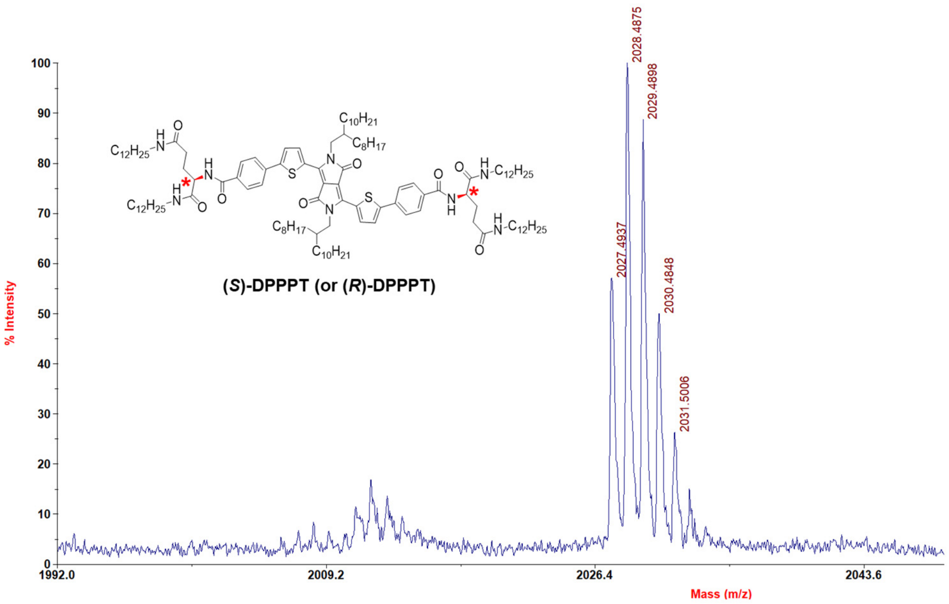

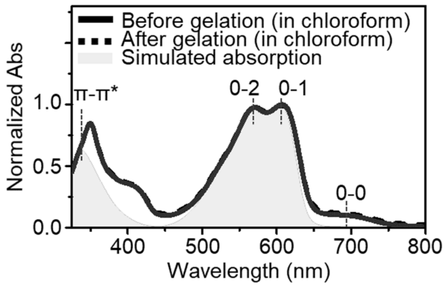

Figure S2. Normalized absorption spectrum of a DPPPT dispersion in chloroform before gelation (dashed line) and after gelation (solid line) the gray regions show the DFT-simulated spectral absorption of DPPPT dimers.

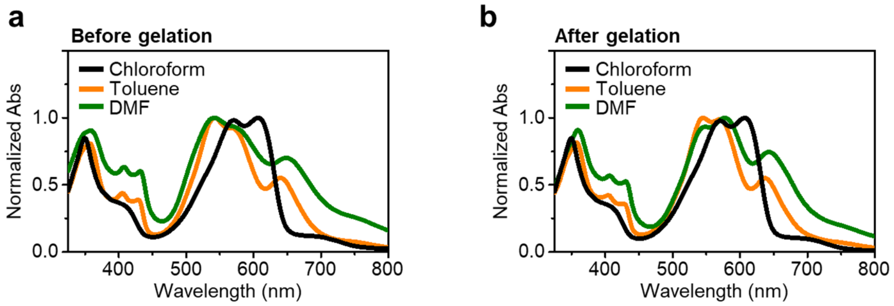

Figure S3. Normalized absorption spectrum of DPPPT dispersed in chloroform (black line), toluene (orange line), and DMF (green line) before gelation (a) and after gelation (b).

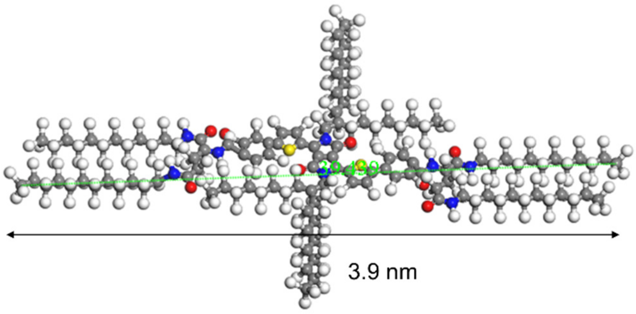

Figure S4. Extended molecular length of DPPPT represented by CPK modeling.

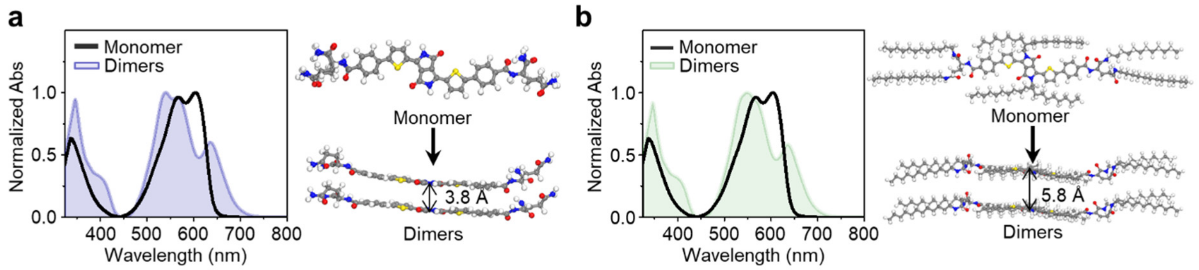

Figure S5. a–b) The DFT-simulated absorption spectrum (left) and calculated intermolecular distance (right) of DPPPT dimers based on a simplified molecular structure without an alkyl side chain on the DPP core (a) and original DPPPT molecular structure (b).

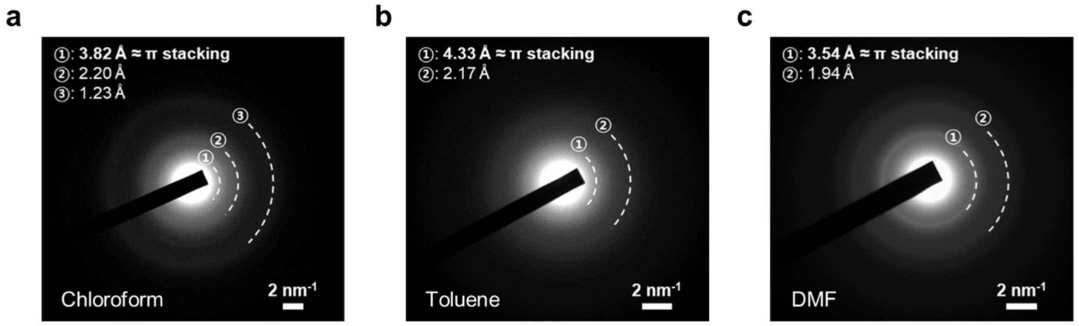

Figure S6. The SAED patterns showing the π–π stacking distance of the drop-cast thin films of the DPPPT assemblies formed in chloroform (a), toluene (b), and DMF (c), respectively.

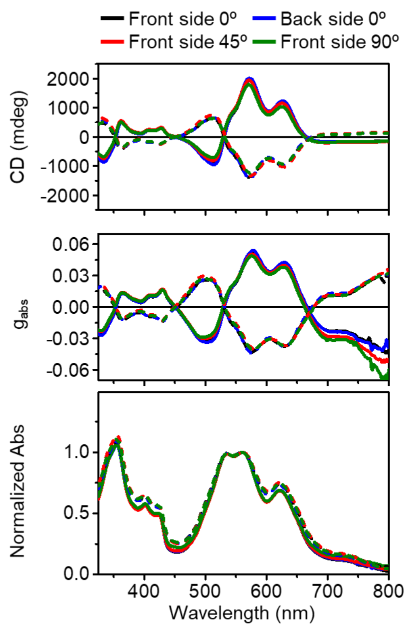

Figure S7. Circular dichroism, dissymmetry factor for absorption (g_{abs}), and normalized absorption spectrum of the drop-cast thin films of the (*R*)-DPPPT (dotted line) and (*S*)-DPPPT (solid line); the measurements were performed from the back or front side of the sample or when the azimuthal angle of the sample was rotated around the optical axis of the incident light.

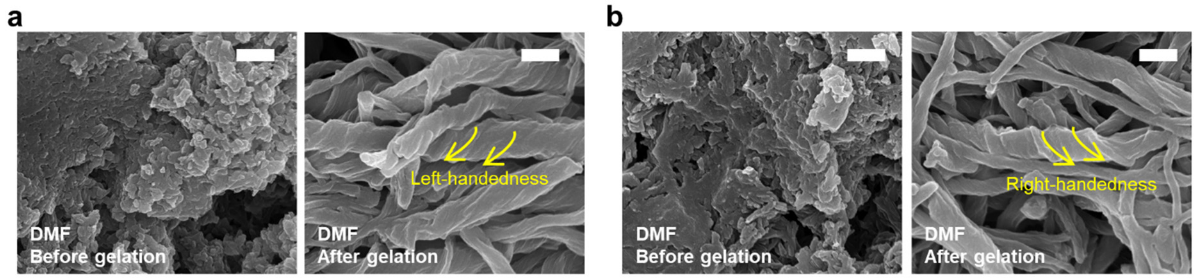

Figure S8. a–b) SEM images of the drop-cast thin films of the (*S*)-DPPPT (a) and (*R*)-DPPPT (b) formed in DMF before gelation (left) and after gelation (right) (scale bar = 500 nm).

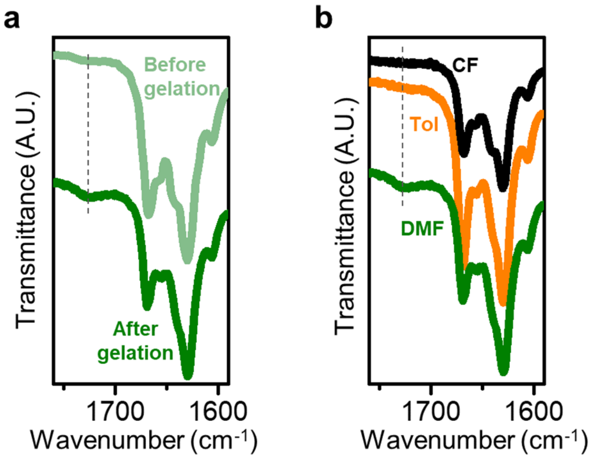

Figure S9. a–b) FT-IR spectrum of the drop-cast thin films of the DPPPT dispersion in DMF before gelation (light-green line) and after gelation (green line) (a), compared to those in chloroform (CF, black line), and toluene (Tol, orange line) after gelation (b).

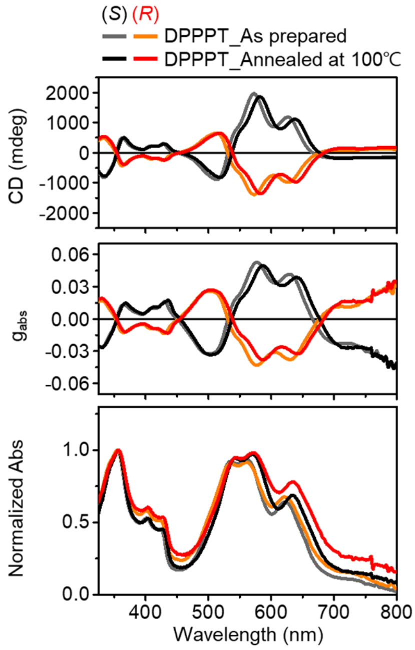

Figure S10. Circular dichroism, dissymmetry factor for absorption (g_{abs}), and normalized absorption spectrum of the drop-cast thin films of (*R*)-DPPPT (red line) and (*S*)-DPPPT (black line) before and after the films were annealed at 100 °C for 30 min.

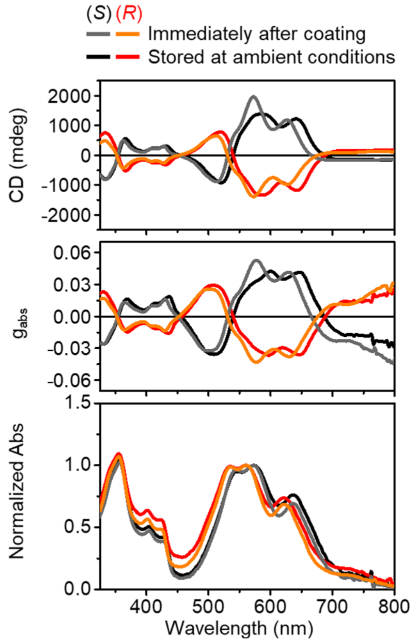

Figure S11. Circular dichroism, dissymmetry factor for absorption (g_{abs}), and normalized absorption spectrum of the drop-cast thin films of (*R*)-DPPPT (red line) and (*S*)-DPPPT (black line) before and after stored in ambient conditions for approximately one year.

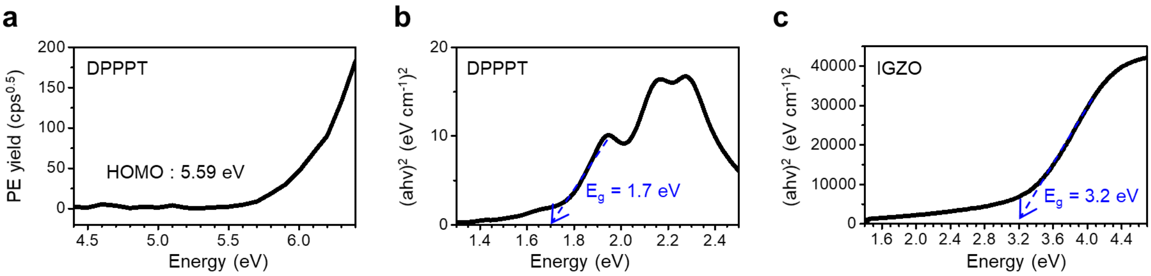

Figure S12. a) Photoemission spectroscopy of DPPPT and b–c) Tauc plots of the DPPPT (b) and pristine IGZO (c).

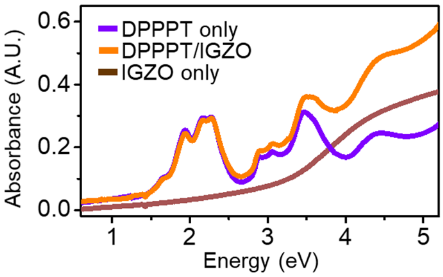

Figure S13. Absorption spectrum of DPPPT (purple), DPPPT/IGZO (orange) and IGZO (brown) thin films.

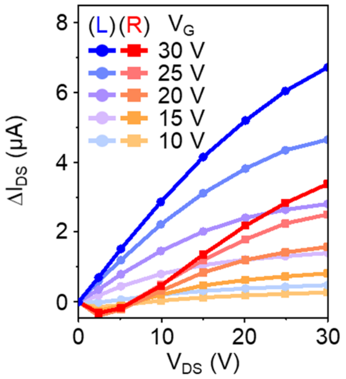

Figure S14. The photocurrent ($\Delta I_{ph} = I_{ph} - I_{dark}$) of (*S*)-DPPPT-cPTr ($10 \leq V_G \leq 30$ V, V_G step = 5 V) irradiation with LCP (blue line) and RCP (red line) light (λ = 520 nm, P_{in} = 500 μW cm^{-2}).

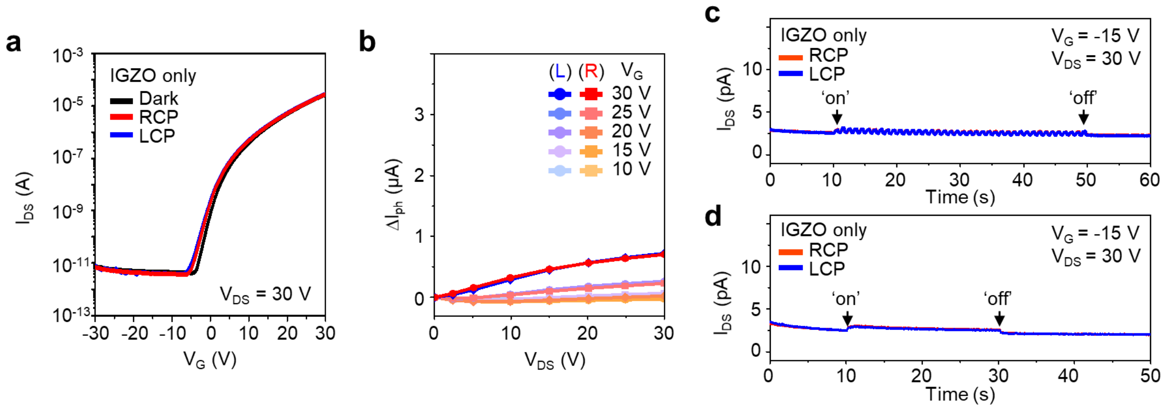

Figure S15. a) Transfer curves of pristine IGZO transistor under dark (black line) and CP light-irradiated conditions (blue and red lines for LCP and RCP light, respectively). A V_{SD} of 30 V was applied to the drain electrode, and a CP light laser (λ = 520 nm, P_{in} = 500 μW cm^{-2}) was used. b) The photocurrents ($\Delta I_{ph} = I_{ph} - I_{dark}$) of pristine IGZO ($10 \leq V_G \leq 30$ V, V_G step = 5 V) upon irradiation with LCP (blue line) and RCP (red line) light (λ = 520 nm, P_{in} = 500 μW cm^{-2}). c–d) Time-dependent photocurrent generation and decay characteristics of pristine IGZO under continuous CP light illumination (duration: 20 s) (c) and under pulsed CP light illumination (duration: 0.5 s, frequency: 1 Hz) (d). At V_{SD} = 30 V, a gate voltage of −15 V was applied and a CP light laser (λ = 520 nm, P_{in} = 500 μW cm^{-2}) was used. Blue and red lines indicate the photoresponse to LCP and RCP light, respectively.

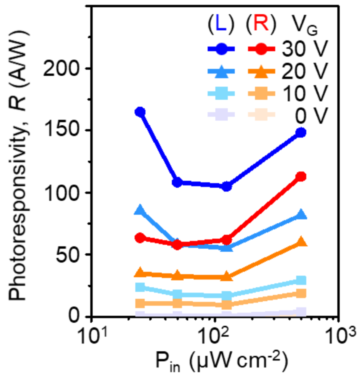

Figure S16. Photoresponsivity of (*S*)-DPPPT-cPTr under irradiation with CP light with different intensities. Gate biases of 0, 10, 20, and 30 V were applied.

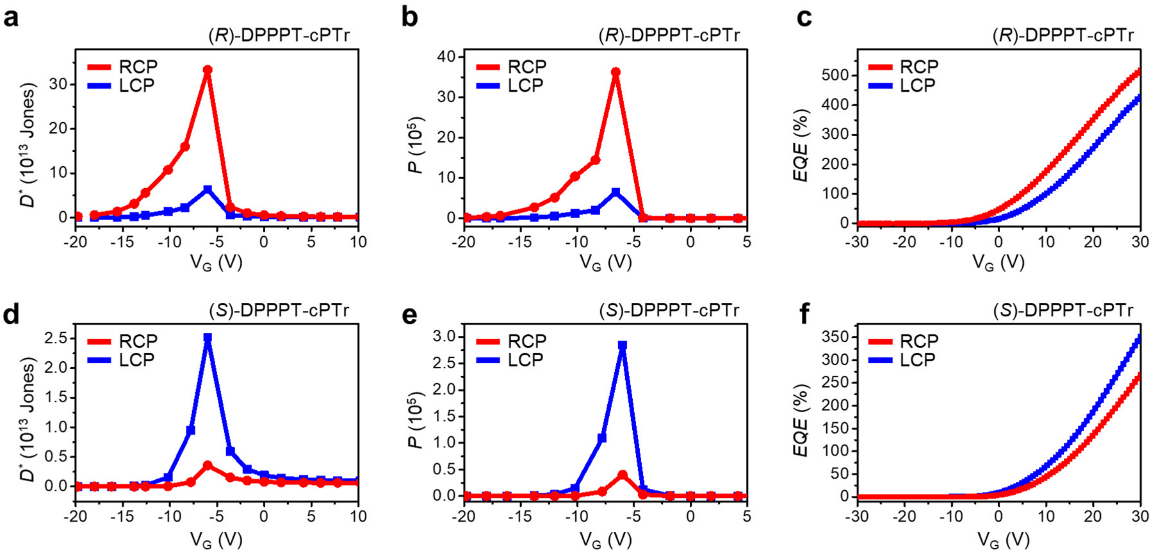

Figure S17. a–c) D^* (a,d), P (b,e), and EQE (c,f) values of (*R*)-DPPPT-cPTr (top) and (*S*)-DPPPT-cPTr (bottom) as a function of V_G under irradiation with CP light (λ = 520 nm, P_{in} = 500 µW cm^{-2}) Blue and red lines indicates the photoresponse by LCP and RCP light, respectively.

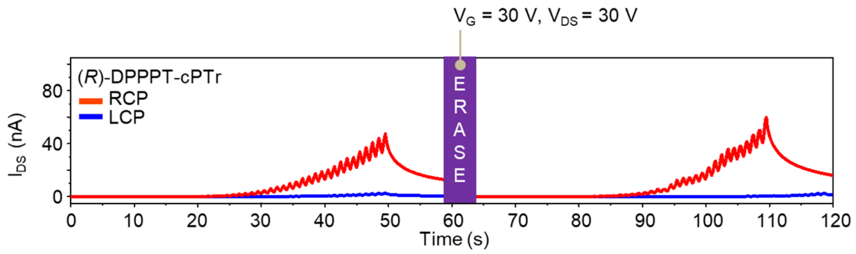

Figure S18. Time-dependent photocurrent generation and decay characteristics of (*R*)-DPPPT-cPTr after a positive gate bias (V_G = 30 V, V_{SD} = 30 V) was applied to recover PPC in response to pulsed CP light illumination (wavelength: 520 nm, P_{in} = 500 µW cm^{-2}, duration: 0.5 s, frequency: 1 Hz). At V_{SD} = 30 V, a gate bias of −25 V was applied to the gate of the (*R*)-DPPPT-cPTr.Blue and red lines indicate the photoresponses to LCP and RCP light, respectively.

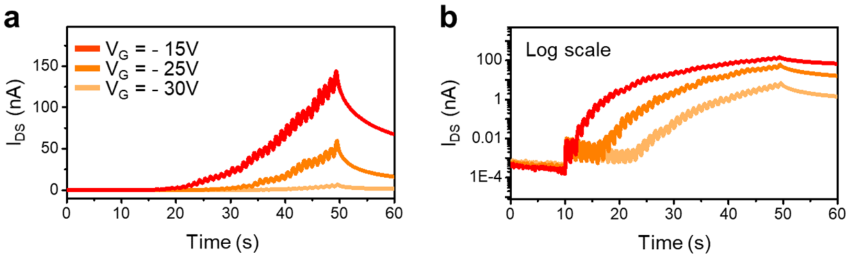

Figure S19. a–b) Log scale (a) and linear scale (b) plots of the time-dependent photocurrent generation and decay characteristics of (*R*)-DPPPT-cPTr depending on the gate bias ($V_G = -15$ V, −20 V, −30 V) at V_{SD} =30 V in response to the pulsed RCP light illumination (wavelength: 520 nm, P_{in} = 500 μW cm^{-2}, duration: 0.5 s, frequency: 1 Hz). The threshold time at which a sudden photocurrent increase occurred was dependent on the applied gate bias.

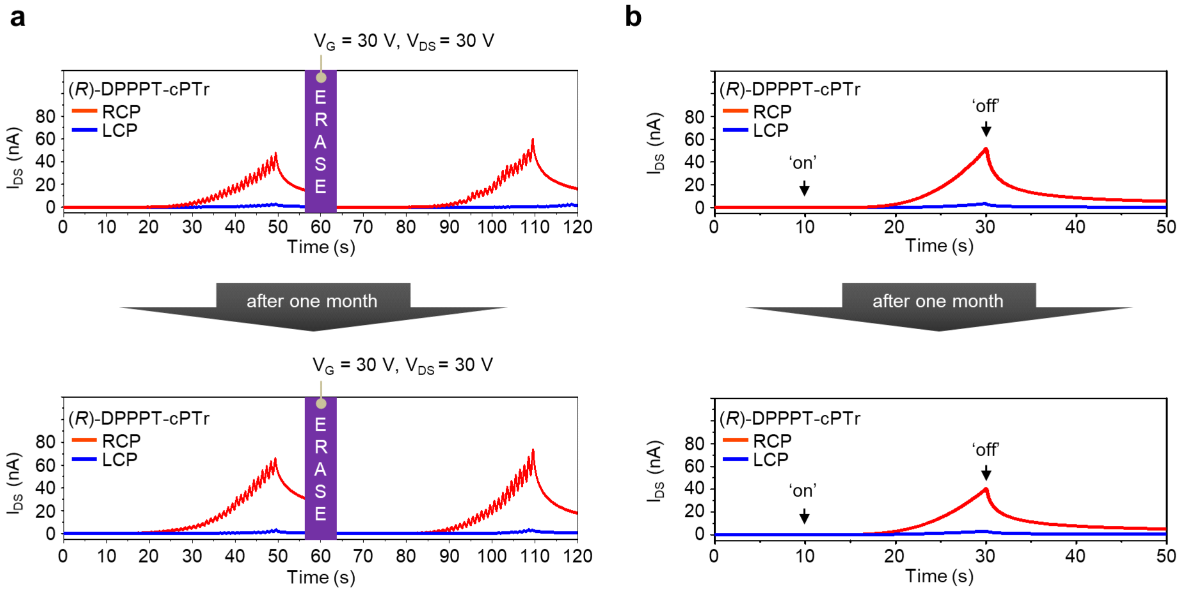

Figure S20. a) Time-dependent photocurrent generation and decay characteristics of (*R*)-DPPPT-cPTr in response to illumination by pulsed CP light (wavelength: 520 nm, P_{in} = 500 μW cm^{-2}, duration: 0.5 s, frequency: 1 Hz) (a) and CP light for 20 s (b) before (top) and after (bottom) storing the DPPPT-cPTr in a vacuum condition for one month. A gate bias of −25 V was applied to the gate of the (*R*)-DPPPT-cPTr. At V_{SD} = 30 V, a gate bias of −25 V was applied to the gate of the (*R*)-DPPPT-cPTr. Blue and red lines indicate the photoresponses to LCP and RCP light, respectively.

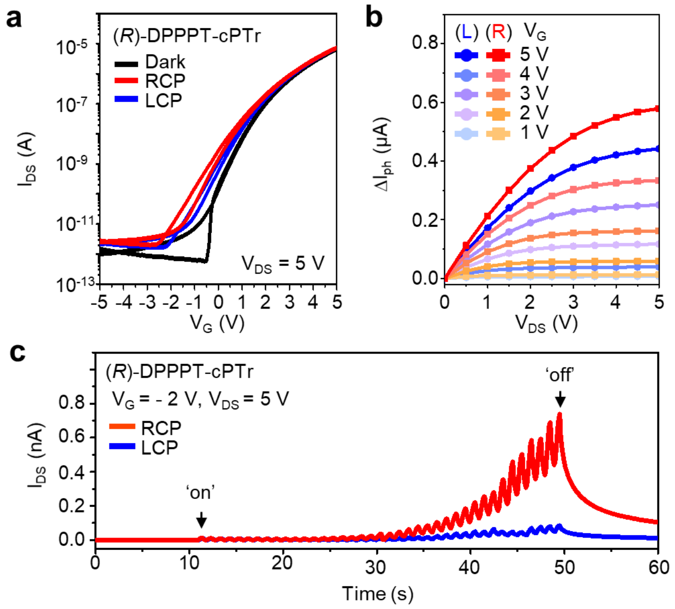

Figure S21. a) Transfer curves of (*R*)-DPPPT-cPTr using the 50 nm-thick Al_2O_3 dielectric under dark (black line) and CP light-irradiated conditions (blue and red lines for LCP and RCP light, respectively), where channel length and channel width are 50 and 1000 μm, respectively. A V_{SD} of 5 V was applied to the drain electrode, and a CP light laser (λ = 520 nm, P_{in} = 500 μW cm^{-2}) was used. b) The photocurrents ($\Delta I_{ph} = I_{ph} - I_{dark}$) of (*R*)-DPPPT-cPTr (10 ≤ V_G ≤ 30 V, V_G step = 5 V) upon irradiation with LCP (blue line) and RCP (red line) light (λ = 520 nm, P_{in} = 500 μW cm^{-2}). c) Time-dependent photocurrent generation and decay characteristics of (*R*)-DPPPT-cPTr in response to illumination by pulsed CP light (wavelength: 520 nm, P_{in} = 500 μW cm^{-2}, duration: 0.5 s, frequency: 1 Hz). At V_{SD} = 5 V, a gate bias of −2 V was applied to the gate of the (*R*)-DPPPT-cPTr device. Blue and red lines indicate the photoresponses to LCP and RCP light, respectively.

Ref.	Chiroptical materials	Device structure	Wavelength (nm)	g_{ph} (or g_R)†	*R* (A/W)	*P*	*EQE* (%)	*D** (Jones)
[1]	[R,S-β-MPA]$_2$MAPb$_2$I$_7$	Perovskite-based diode	532	g_{ph} : 0.2	3.8		882	1.1×10^{12}
[2]	(R,S-NEA)$_2$PbI$_4$	Perovskite-based diode	395	g_{ph} : 1.84	0.28	-	88	-
[3]	ClCPDI-Ph-CF	Organic field-effect transistor	495	g_R : 0.13, -0.12	1129	1.7×10^{7}	300000	2.2×10^{16}
[4]	Aza[6]helicene	Organic field-effect transistor	365	g_{ph} : 1.8	-	-	-	-
[5]	3a-P or M	Organic field-effect transistor	635<br>730	g_R :<br>–0.054, 0.057<br>0.01, –0.009	0.45	2.5	89	2.1×10^{10}
[6]	F6BT:P3HT	Organic field-effect transistor	450	g_R : 1.92, -1.94	1.9×10^{-5}	40.3	0.012	7.4×10^{7}
[7]	Chiral Ag/Si	Heterojunction schottky diode	1350	g_R : 1.09	2.2×10^{-3}	-	-	-
[8]	(R)-1 or (S)-1/P3HT	Heterojunction transistor	450	g_R : 0.83, -0.82	3.52	7.8	968	1.0×10^{12}
[9]	Au NP/IGZO	Heterojunction transistor	780<br>635	-	6.50	-	-	2.0×10^{10}
**This work**	DPPPT/IGZO	Heterojunction transistor	520	g_R : ±1.97<br>g_{ph} : ±1.97	218	3.6×10^{6}	518	3.3×10^{14}

Table S1. Comparison of the chiroptical photodetection parameters of DPPPT/IGZO chiroptical synaptic heterojunction phototransistor compared to those of previously reported chiroptical photodetectors fabricated using various nanomaterials. †g_{ph} and g_{R} denote the dissymmetry factors calculated by using photocurrent and responsivity, respectively.

† $g_{\mathrm{ph}} = 2(I_{\mathrm{ph}}^{\mathrm{L}} - I_{\mathrm{ph}}^{\mathrm{R}})/(I_{\mathrm{ph}}^{\mathrm{L}} + I_{\mathrm{ph}}^{\mathrm{R}})$, $g_{\mathrm{R}} = 2(I_{\mathrm{R}}^{\mathrm{L}} - I_{R}^{\mathrm{R}})/(I_{\mathrm{R}}^{\mathrm{L}} + I_{\mathrm{R}}^{\mathrm{R}})$, where $I_{\mathrm{ph}}^{\mathrm{L}}$ and $I_{\mathrm{ph}}^{\mathrm{R}}$ are the photocurrents induced by LCP and RCP light, respectively, and $I_{\mathrm{R}}^{\mathrm{L}}$ and $I_{\mathrm{R}}^{\mathrm{R}}$ are the photoresponsivity induced by LCP and RCP light, respectively.

References

[1] L. Wang, Y. Xue, M. Cui, Y. Huang, H. Xu, C. Qin, J. Yang, H. Dai, M. Yuan, *Angewandte Chemie* **2020**, *132* (16), 6504.
[2] A. Ishii, T. Miyasaka, *Science advances* **2020**, *6* (46), eabd3274.
[3] X. Shang, I. Song, J. H. Lee, W. Choi, J. Ahn, H. Ohtsu, J. C. Kim, J. Y. Koo, S. K. Kwak, J. H. Oh, *ACS nano* **2020**, *14* (10), 14146.
[4] Y. Yang, R. C. Da Costa, M. J. Fuchter, A. J. Campbell, *Nature Photonics* **2013**, *7* (8), 634.
[5] L. Zhang, I. Song, J. Ahn, M. Han, M. Linares, M. Surin, H.-J. Zhang, J. H. Oh, J. Lin, *Nature Communications* **2021**, *12* (1), 142.
[6] J. Cheng, F. Ge, C. Zhang, Y. Kuai, P. Hou, Y. Xiang, D. Zhang, L. Qiu, Q. Zhang, G. Zou, *Journal of Materials Chemistry C* **2020**, *8* (27), 9271.
[7] W. Li, Z. J. Coppens, L. V. Besteiro, W. Wang, A. O. Govorov, J. Valentine, *Nature communications* **2015**, *6* (1), 8379.
[8] Y. Xue, C. Zhang, T. Lv, L. Qiu, F. Wang, *Angewandte Chemie International Edition* **2023**, e202300972.
[9] S. D. Namgung, R. M. Kim, Y.-C. Lim, J. W. Lee, N. H. Cho, H. Kim, J.-S. Huh, H. Rhee, S. Nah, M.-K. Song, *Nature Communications* **2022**, *13* (1), 5081.
